# Supplementary material for: Developing a recovery-focused therapy for older people with bipolar disorder: a qualitative focus group study
Source: BMJ Open. 2021 Aug 4;11(8):e049829. doi: 10.1136/bmjopen-2021-049829 (PMC8340279; doi:10.1136/bmjopen-2021-049829)
Supplement: Supplementary data [file bmjopen-2021-049829supp002.pdf]

Focus group topics and content

Focus group 1

1. Icebreaker

Focus group 1 started with an icebreaker (participants went around the room introducing themselves and telling the group an interesting fact about themselves).

2. Recovery approach introduced

The recovery approach was introduced and handed out on a piece of paper, defined as:

"Recovery is being able to live a meaningful and satisfying life, as defined by each person, in the presence or absence of symptoms. It is about having control over and input into your own life. Each individual's recovery, like his or her experience of the mental health problems or illness, is a unique and deeply personal process." (Scottish Recovery Network)

Participants were asked to comment on:

- their thoughts about this quote
- the term 'recovery'
- what recovery meant to them in later life

3. Experience of living with bipolar disorder in later life

Participants were asked to talk about their experience of living with bipolar disorder in later life.

4. Ways of coping with bipolar disorder in later life

Participants were asked about different ways of coping with the condition (including support from relatives and health professionals) in later life.

Focus group 2

1. Introductions

Focus group 2 started with everyone introducing themselves again, with the chance for new participants to tell an interesting fact about themselves.

2. Introducing recovery focused CBT and the stages of therapy

Recovery focused CBT therapy was introduced to the participants and the stages of therapy were discussed:

1. Introducing the recovery approach to clients;
2. Collection of information about current and historical mood and functioning;
3. Meaning and relevance of diagnosis;
4. Identification of recovery-informed therapy goals;
5. Initial formulation of relationships between mood experiences and progress towards recovery goals;

6. Identification and application of CBT techniques to address and facilitate positive coping;
7. Consideration of wider functioning issues in relation to recovery;
8. Development and completion of early warning signs (EWS) plan;
9. Development and completion of recovery plan;
10. Sharing lessons from therapy with key stakeholders.

Participants were asked to comment on:

- whether they thought the approach would be helpful with older adults
- what the strengths and weaknesses of the approach were
- what modifications (if any) would be needed for an older population

2. Session structure

Participants were told that the intervention would be delivered in one-to-one sessions and asked:

- what else might be important to include in therapy sessions for older people
- what might make therapy more accessible and effective for older people

3. Experience of therapy

Participants were asked to comment:

- if they had experience of receiving psychological therapy
- what they had found helpful/ unhelpful when accessing care and support (e.g. CPN, psychiatrist, therapist, relative) in the past
- what help they would want in the future

Focus group 3

1.Introductions

Focus group 3 started with everyone introducing themselves again.

2. Topics

All of the topics above were re-visited in focus group 3 so the three participants who hadn't attended 1 or 2 were able to share their ideas.
