# Supplementary material for: Developing a recovery-focused therapy for older people with bipolar disorder: a qualitative focus group study
Source: BMJ Open. 2021 Aug 4;11(8):e049829. doi: 10.1136/bmjopen-2021-049829 (PMC8340279; doi:10.1136/bmjopen-2021-049829)
Supplement: Supplementary data [file bmjopen-2021-049829supp003.pdf]

Theme	Example quotes
Health and age-related changes in later life	"My concentration isn't very good and the sorts of books I read are different. I can't read anything particularly frightening"<br>"I'm a bit slower but yeah, my memory is OK but like as soon as I get depressed, I get even worse"<br>"When my moods alright so it's slightly elevated, I am … on the ball. I can remember anything…as soon as my mood starts to dip then I start to not…I can't remember what I did the day before"<br>"I think my mood has an effect on my memory…it just gets hard as you get older"<br>"Just my ability to remember things. It's in a limited way now. I put it down to the medication"<br>"I think the only thing I actually do is to listen to the radio and read a certain amount"<br>"We get fed up and we get frustrated and all those things because we can't do what we did"<br>"As you get older there's a lot more…quite often you've got more health issues you've got to deal with...those things can isolate XXX quite a lot. So, when she feels more isolated it can affect mood"<br>"Family members go and social situations change and health deteriorates"<br>"Family structure does change…it's partly what upsets me, I suppose, when I'm in a low. You know, you look back...how many friends and how many family…have gone and you think Christ, you know, I'm the only one here. You don't expect to be the last one."<br>"Such a lot gone. And you just think I miss some of them around me and I must get what I want to do done"

	"The peer thing reduces when you get older... I think the thing that loses, if you get a good peer situation, your identity…when your older, there's a little bit of a problem trying to find your identity".<br>"You're lonely and perhaps not as sharp as you were. So maybe it's a little bit more difficult…maybe we want it but how do we go about it. How do I go about getting involved back in life again"
Experience of BD in later life	"I found that with my manic episodes it's taken me less time to get stable again as the years have gone by. So whether that's an indication that in later life that they will be able to recover quicker and they're not as severe, I don't know."<br>"It counts up to about eight whereas before it went up to nine"<br>"I'm mostly downers. Well if I get in the middle of a picture I'm painting and I'm like enjoying it, I get a high because it's made me really happy…but most of it…I go quiet"<br>"As the depressions have got worse everything pretty much shuts down and I'm depressed"<br>"When I'm on a high I feel like my minds looking up…but when I'm down, I feel like my mind's closed"<br>"I think it loses itself because you can't do the things you did…I do sometimes, and I'm sure everyone does, feels as if well, who am I now? What have I got to contribute?"<br>"Launching yourself out was no problem when you're young, you know, because confidence"<br>"You've got to believe in yourself and you have low self-esteem when you've got bipolar. And people think because you are mentally ill…you need you need to be treated differently"

	"I've got bipolar but I don't go telling other people because I know that, particularly my age group…if you're got to declare a mental illness, it's like well you're not one of them"<br>"It may be that...my psychological problems are sort of focused around get worse as they don't get solved or accepted all the time"<br>"Coming to terms with events that have happened, and you can't understand why that happened and why you did that and why you did this. And with all the embarrassment to cope with"<br>"Yeah, it's regret. Wishing that I'd been, behaved differently or treated somebody differently and that"<br>"If you're doing something that doesn't require a lot of concentration, these things come back to the mind"<br>"I think what's made a difference for my, for my mistakes is that I do have a living faith in God whom I can turn"<br>"What you've got to remember is that if you've got bipolar all your life you've got secrets…you've got personal secrets"<br>"And she kind of knows what she needs to do but there's a greater sense of hopelessness which might be just to do with less life span"
Managing and coping with BD in later life	"There's also been a change in, from my limited experience, that the medications seem to be only a, you know, pop a few pills.... that was the limit of it a while ago but there seems to be other areas being examined ...for example what you are doing"

	"I personally am terrified about what the medication – all the medication I'm on is going to do to me twenty years down the line... what effect it's going to have on my brain"<br>"As you grow older you don't need the amount of medication that you had when you were younger"<br>"Because of the medication being a lot more stable...Ive been able to undertake duties"<br>"I'm able to stabilise myself, sort of consciously change, lifestyle changes, depending on what mood I'm in... act on it quickly and so that I can reach a level ground again"<br>"I think it's a case of learning the illness and being able to manage the illness"<br>"I've painted a lot of pictures which is of course therapeutic"<br>"I think we are capable of doing an awful lot that people think we can't do and therefore we tend to play down our expectations and don't achieve our full potential...if people encourage us, it's surprising wheat we can achieve".<br>"It's about setting out the right goals as well...what older people might be capable of and talking about the sort of things that might be in our own heads individually and whether they are achievable".<br>"If you're having a good weeding session on the allotment, you're concentrating on what you're doing but if you're doing something that doesn't require a lot of concentration, these things come back to mind".<br>"I actually need to be doing at least two things to have a chance of distracting myself. So one way I get through the day when I'm low is to have the radio on but I'm also reading something"

	“I’m in charge of health and safety... and it, well it keeps me mind occupied doesn’t it?<br><br>“I would expect health professionals to help you to bond to that community. I would expect them to know what is available to you, to signpost you”<br><br>“As you get older you get more assertive with your professionals”<br><br>“There is a difference now that the professionals treat me with respect and they ask my opinion and how often I want to see them”<br><br>“The younger doctors that I’ve had dealings with have been brilliant – they've been entirely professional but they’ve also been informal”<br><br>We’re still fighting professionals who don’t believe that we’re capable of what we truly are and so we don’t realise our full potential”<br><br>“A lot of older people they often had a partner that’s helped kind of co-helped them co-manage the illness. And when that partner goes, so when my relative died... I didn’t realise at the time but he helped modify”<br><br>“I’ve got a friend now...she phones me up in the morning at eight o’clock and says, how are you and we can be honest with one another”

Experience of recovery in later life	"This recovery we're just talking about... It's something that I look beyond the horizon"<br>"I think the word recovery... you aim for recovery...I think it should be called stability, not recovery"<br>"I don't think you ever recover...you manage it"<br>"Recovery for my therapy team was for me not to go back into hospital... But for me recovery was I really wanted to get another job. It was really important to me"<br>"I'm not sure at my age just to what degree I can move on really as time is flying by… I've been told you can never recover from bipolar"<br>"There is a great difficulty with the word recovery with professionals in the past by they are coming round"<br>"I'm a member of various organisations...I've got an activity every single day to do. And this helps... I'm out and about and I'm mixing with people and this helps enormously".<br>"I think It's possible to be recovered and I reckon that I am recovered now...I have the right medication...and I self-manage. I have a healthy lifestyle and I meet a lot of people"<br>"I've still got a life to live...I can meaningfully contribute to society"<br>"I want to move on from this. There's something better than this. If we haven't got hope, we're just going to wallow"<br>"You wouldn't want to recover if you didn't have hope"

Seeking help in the future	“I was thinking of having time round the therapy... having more time with an old person, sort of fifteen minutes before and fifteen minutes after... just being human together because it’s hard doing therapy anyway”<br>“It’s about having that personal relationship. It takes two or three sessions to get the confidence”<br>“I think as you get older, you perhaps become quicker at knowing whether you’re going to click with somebody”<br>“And we’re not as impressable”<br>“We can’t be fooled too easily, can we?”<br>“They need to have listening skills. They need to be adapting body language and tone of voice and pitch of voice. They need to be empathic. And they need to be aware of the sort of problems that we face. And they need to get to the cause”<br>“You could raise the gender thing as an explicit issue... find out whether the client has any issues about the gender of the therapist”<br>“The therapist should try to make you feel assertive. Train you to be assertive”<br>“It’s part of the therapist's duty to give you higher autonomy and assertiveness so you can fight your corner in a non-aggressive way but one that is assertive”<br>“Therapy focused partly on older age needs to help people to improve competence in whatever area they wanted”<br>“I think that could be a bit of a stumbling block for older people to be able to talk to younger people”

Adapting RfCBT for older people	"I think the idea of fifty minutes is partly that you can have a little bit of chant, you know, at the beginning. Sort of hello, how are you".<br>"When you get older, you know, with sight and problems and hearing problems you just need more resources...things written down... sort of more back up"<br>"She can't take lots of information in really quickly. So more time. Writing things down. Get her to write it down"<br>"Can you break it down...into simple clear language"<br>"Some large sort of text...if it's complicated it could be simplified"<br>"You need to use more images"<br>"It's more interesting to kind of ...engage with and it's easy to kind of, like you said about remembering things you have something to hang on to"<br>"I think a video would be extremely useful...then you can … a disk that you can put in computer and you could watch on the screen an instruction"<br>"If you have it once a fortnight, you've time to put into practice what's been said and what's been taught"<br>"It's quite useful to have a gap...you've learnt some practical strategies...then you've had time to practice on your own...without the weekly backup"<br>"The idea of a short-ish course and then maybe going back in"
