# Supplementary material for: Developing a recovery-focused therapy for older people with bipolar disorder: a qualitative focus group study
Source: BMJ Open. 2021 Aug 4;11(8):e049829. doi: 10.1136/bmjopen-2021-049829 (PMC8340279; doi:10.1136/bmjopen-2021-049829)
Supplement: Author's manuscript [file bmjopen-2021-049829.draft_revisions.pdf]

BMJ Open is committed to open peer review. As part of this commitment we make the peer review history of every article we publish publicly available.

When an article is published we post the peer reviewers' comments and the authors' responses online. We also post the versions of the paper that were used during peer review. These are the versions that the peer review comments apply to.

The versions of the paper that follow are the versions that were submitted during the peer review process. They are not the versions of record or the final published versions. They should not be cited or distributed as the published version of this manuscript.

BMJ Open is an open access journal and the full, final, typeset and author-corrected version of record of the manuscript is available on our site with no access controls, subscription charges or pay-per-view fees (http://bmjopen.bmj.com).

If you have any questions on BMJ Open's open peer review process please email info.bmjopen@bmj.com

BMJ Open

Developing a recovery-focused CBT therapy for older people with bipolar disorder: A qualitative focus group study

Journal:	*BMJ Open*
Manuscript ID	bmjopen-2021-049829
Article Type:	Original research
Date Submitted by the Author:	04-Feb-2021

Complete List of Authors:	Tyler, Elizabeth; Lancaster University, Lancaster University Division of Health Research<br>Lobban, Fiona; Lancaster University, Lancaster University Division of Health Research<br>Long, Rita; Lancaster University, Lancaster University Division of Health Research<br>Jones, Steven; Lancaster University, Lancaster University Division of Health Research

Keywords:	MENTAL HEALTH, GERIATRIC MEDICINE, Depression & mood disorders < PSYCHIATRY

SCHOLARONE™
Manuscripts

I, the Submitting Author has the right to grant and does grant on behalf of all authors of the Work (as defined in the below author licence), an exclusive licence and/or a non-exclusive licence for contributions from authors who are: i) UK Crown employees; ii) where BMJ has agreed a CC-BY licence shall apply, and/or iii) in accordance with the terms applicable for US Federal Government officers or employees acting as part of their official duties; on a worldwide, perpetual, irrevocable, royalty-free basis to BMJ Publishing Group Ltd ("BMJ") its licensees and where the relevant Journal is co-owned by BMJ to the co-owners of the Journal, to publish the Work in this journal and any other BMJ products and to exploit all rights, as set out in our licence.

The Submitting Author accepts and understands that any supply made under these terms is made by BMJ to the Submitting Author unless you are acting as an employee on behalf of your employer or a postgraduate student of an affiliated institution which is paying any applicable article publishing charge ("APC") for Open Access articles. Where the Submitting Author wishes to make the Work available on an Open Access basis (and intends to pay the relevant APC), the terms of reuse of such Open Access shall be governed by a Creative Commons licence – details of these licences and which Creative Commons licence will apply to this Work are set out in our licence referred to above.

Other than as permitted in any relevant BMJ Author's Self Archiving Policies, I confirm this Work has not been accepted for publication elsewhere, is not being considered for publication elsewhere and does not duplicate material already published. I confirm all authors consent to publication of this Work and authorise the granting of this licence.

For peer review only

Developing a recovery-focused CBT therapy for older people with bipolar disorder: A qualitative focus group study

Dr Elizabeth Tyler : Lancaster University Division of Health Research, Lancaster, UK

Professor Fiona Lobban : Lancaster University Division of Health Research, Division of Health Research, Lancaster University, Lancaster, UK

Ms Rita Long: Lancaster University Division of Health Research, Lancaster University, Lancaster, UK

Professor Steven Jones : Lancaster University Division of Health Research, Lancaster University, Lancaster, UK

Corresponding author: Dr Elizabeth Tyler: e.tyler@lancaster.ac.uk

For peer review only

For peer review only - http://bmjopen.bmj.com/site/about/guidelines.xhtml

ABSTRACT

Objectives: As the world's population ages and there is increased awareness of Bipolar Disorder (BD) the number of older people living with the condition will rise substantially. There is no current evidence base for the effectiveness of psychological interventions for older adults with BD. This focus group study explored a number of topics to inform the development and delivery of a recovery-focused cognitive behavioural therapy (RfCBT) for older adults in a feasibility randomised controlled trial.

Design: A qualitative focus group study.

Setting: Three focus groups were conducted at a University in the North-West of England.

Participants: Eight people took part in the focus groups; six older adults with BD, one carer and one friend.

Results: Participants responses clustered into 6 themes: 1) health and age-related changes in later life, 2) the experience of BD in later life, 3) managing and coping with BD in later life, 4) recovery in later life, 5) seeking helping in the future 6) adapting RfCBT for older people.

Conclusions: Participants reported a range of health and age-related changes and strategies to manage their BD. Participants held mixed views about using the term 'recovery' in later life. Participants were in agreement that certain adaptations were needed for delivering RfCBT for older adults, based upon their experience of living with BD in later life. The data collected as part of the focus groups has led to a number of recommendations for delivering recovery focused CBT for older adults with bipolar disorder.

For peer review only - http://bmjopen.bmj.com/site/about/guidelines.xhtml

ARTICLE SUMMARY

Strengths and limitations of this study

To our knowledge this is the first study to involve older adults with BD to shape and develop a psychological intervention specifically for their cohort.

The study was designed and conducted in consultation with service user representation throughout, enhancing the quality, value and the relevance of the study.

The clinical recommendations for delivering RfCBT for older adults have been developed in partnership with individuals with lived experience of BD, carers and healthcare professionals.

The participants taking part in the group were not representative of all older people with bipolar disorder. They were all White British and all apart from one had (or had retired from) a professional working background.

For peer review only

For peer review only - http://bmjopen.bmj.com/site/about/guidelines.xhtml

BACKGROUND

Approximately 25% if individuals living with Bipolar Disorder (BD) are age 60 or over (1). As the world's population ages (2) and awareness of the condition increases, the number of people living with BD into later life is expected to rise substantially. Research indicates that older adult specific services are better placed to meet the needs of those with mental health problems in later life, when compared to general adult services (3). At present, there is no evidence base for the effectiveness of psychological interventions for older adults with BD. The National Institute for Health and Care Excellence BD guideline (4) suggests that older adults with BD should be offered the same treatments as younger people, however there appear to be unique, clinical characteristics that feature in the older population (5), that may impact on their needs and response to treatment (6).

The aim of the programme of work was to adapt recovery focused CBT (RfCBT) intervention developed for younger adults (7), so it could be offered to people with BD over the age of 60. The focus group work sits within a larger study which consists of two phases (see Tyler et al, 8 for trial protocol paper). Phase 1 focused on the development of the recovery focused intervention. Informed by a) the existing recovery focused CBT manual (7) b) a review of the literature of psychological interventions adapted for older adults with other mental health problems (e.g., 9, 10), c) data from the 3 focus groups of older adults with BD and relatives/ friends, d) the expert knowledge from our research team. Phase 2 is an ongoing randomised controlled trial to assess the feasibility and acceptability of recovery focused CBT for older adults with BD (ISRCTN registry: ISRCTN13875321; 8). The key aims of the focus groups were to: (1) explore the extent to which the original RfCBT intervention was acceptable to older adults with BD, (2) identify whether any adaptations would be needed to the existing manual and what support older adults would want from a therapist during therapy, (3) explore the experience of BD in later life; including the relationship with relatives and health professionals and the concept of recovery with BD. All of this

For peer review only - http://bmjopen.bmj.com/site/about/guidelines.xhtml

information has informed the development and the delivery of the RfCBT for older adults in phase 2. This paper reports the results from the focus group work.

METHOD

Design

The study was approved by the UK NHS Ethics Committee process (REC ref: 15/NW/0330). The British Medical Journal (BMJ) and the Medical Research Council (MRC) recommend the incorporation of qualitative research in the process of complex intervention development (11, 12). Focus group methodology (13) was chosen because the researcher was interested in understanding the topics from a diverse range of perspectives, moderating the discussion from a peripheral role (14). The Standards for Reporting Qualitative Research (SRQR) were adhered to and a copy of the checklist can be found in the supplementary information (Supplementary file 1).

Patient and public involvement

Patients and public involvement (PPI) representatives were involved in the study design process from the outset. The team has a service user advisory panel, led at the time by RL. The panel provided feedback on the original grant application and reviewed study documents, including participant information sheets, consent forms and topic guides. At the end of the focus group, participants were invited to remain in the study and form the service user reference group (SURG) to contribute towards the design, implementation and dissemination at phase two.

Sampling and recruitment

Participants were recruited via a confidential database of individuals who have previously consented being approached about potential involvement in research studies. To be eligible to take part in the study, participants were required to identify themselves as an older adult with BD or be a relative or a friend of an older adult with BD (to offer a diverse range of perspectives), have the capacity to

For peer review only

For peer review only - http://bmjopen.bmj.com/site/about/guidelines.xhtml

provide informed consent and have sufficient English language skills to read the information sheet and take part in the discussions.

We aimed to recruit approximately 6-12 participants to take part in the focus groups, consistent with focus group methodology (13, 15, 16). All interested individuals were invited to attend each of the 3 focus groups.

Topic guide for focus groups

A topic guide was developed with the research team (ET, SJ, FL and RL) and designed to loosely structure the focus group and lead the discussion. See supplementary file 2 for focus group topics and content. Different topics were explored in groups 1 and 2 (see table 1) and during group 3 the researcher re-visited all 6 topics to gather more rich and detailed information and ensure that all participants taking part had the opportunity to express their ideas on each of the areas. See table 2 for who attended each group.

Focus group	Topics explored
1	1. Overview of proposed therapy<br>2. Living with BD in later life<br>3. Experience of recovery in later life
2	4. Adapting RfCBT for older people<br>5. What support people want from a therapist during therapy<br>6. Relationship with relatives and health professionals
3	1. ALL Topics re-visited

Table 1: Focus group topics

For peer review only - http://bmjopen.bmj.com/site/about/guidelines.xhtml

For peer review only

Procedure

The groups were conducted at a University in the North-West of England and lasted for approximately 90 minutes, consistent with focus group methodology (13). The first focus group was facilitated by two members of the research team (a service user researcher and the lead researcher). The service user researcher not able to groups 2 and 3 which were facilitated by the lead researcher alone. All groups were audio recorded, and transcribed prior to analysis. All participants provided written informed consent.

ANALYSIS

The focus groups were transcribed and analysed thematically using framework analysis (17), a popular way to analyse primary qualitative data in the area of healthcare (18). This allows for both deductive and inductive coding, with concepts or themes identified as coding categories *a priori* to be combined with other themes that emerge *de novo* (18). Five topics formed the original framework; living with BD in later life, experience of recovery in later life, adapting RfCBT for older people, what support people want from a therapist during therapy and the relationship with relatives and health professionals

Furber (19) identified five phases of framework analysis; familiarisation, a theoretical framework, indexing, charting and synthesis. The three transcripts were read and re-read by the lead researcher (ET) to aid the process of familiarisation, before undertaking the initial coding for transcript 1. Two members of the research team (SJ and FL) independently read and coded (indexed) transcript 1. A meeting took place within the team (ET, SJ and FL) to discuss the transcript, why the coded sections had been interpreted as meaningful and to discuss new codes and the development of the theoretical framework. The new framework was then applied to transcripts 2 and 3. Team meetings took place to discuss any further amendments to the framework, based on the emergence of new

For peer review only

codes. The final framework was a representative of the entire data set collected from the 3 focus groups.

Reflexivity

The researcher was aware that they (and other members of the research team) were approaching the study with a set of pre-conceived ideas about the recovery focused approach and the direction of therapy. SJ developed the original recovery focused CBT manual for an adult BD population. SJ and FL were involved in a recently completed a trial exploring its feasibility and acceptability (8). Subsequently ET, SJ and FL were involved in a case series looking at the application of this approach for individuals with a more established BD diagnosis. The researcher aimed to facilitate the discussion regarding these topics in an open fashion to ensure that the participants felt able to give open and honest feedback about the approach. Similarly, when analysing and interpreting the results, the role of potential bias was highlighted to ensure that the results were a true reflection of the participant's ideas, rather than the team selecting the responses which aligned with their personal view on the topic.

RESULTS

Participants

All participants were invited to attended each focus group; however, this was not achievable due to their various commitments and therefore we were flexible in response to their availability. As shown in table 2, eight participants took part across the 3 focus groups attending 1-3 groups each; 6 service users and 1 carer and 1 friend (who also had a diagnosis of BD and identified herself as moving into later life). Participants ages ranged from 48 to 77 years (M = 66.1). Participants with BD had an established diagnosis, ranging from 8 to 38 years. All of the sample were White British and the majority were female (n= 5; 63%). The majority of the sample were retired (n=5; 63%) and the majority had professional backgrounds either currently or prior to retirement (n = 7; 88%). The

For peer review only

For peer review only - http://bmjopen.bmj.com/site/about/guidelines.xhtml

majority of the sample had experience of psychological therapy (n=6; 75%), with (n=4; 50%) of participants receiving CBT in the past.

Table 2: Participant characteristics

REDACTED

Themes

There was a great deal of rich data regarding how participants described perceived changes during their later years. Once the team met to code focus group 1, using a combination of both inductive and deductive analysis, they agreed that the framework should be revised to reflect the data. The original topic “living with BD in later life” was broadened to encompass 3 themes; “health and age-related changes in later life”, “experience of BD in later life”, “managing and coping with BD in later life”, following and inductive approach. Initial topics of interest “relationship with relatives and health professionals” became subthemes within the new theme “managing and coping with bipolar in later life”. Original topic “what support people want from a therapist” became a new theme “seeking helping the future”. The original topics of interest “experience of recovery in later life” and “adapting RfCBT for older people” became the final themes, following a deductive approach. Please see table 3 for the preliminary and final themes. Additional example quotes can be found in supplementary file 3.

For peer review only - http://bmjopen.bmj.com/site/about/guidelines.xhtml

PRELIMINARY THEME	FINAL THEME	SUB-THEME
**LIVING WITH BD IN LATER LIFE**	**HEALTH AND AGE-RELATED CHANGES IN LATER LIFE**	Cognitive symptoms<br>Behavioural changes<br>Physical symptoms<br>Social network changes<br>Loneliness and isolation
**LIVING WITH BD IN LATER LIFE**	**EXPERIENCE OF BD IN LATER LIFE**	Change in psychological symptoms<br>Identity<br>Stigma<br>Guilt and shame
**LIVING WITH BD IN LATER LIFE**<br>**RELATIONSHIP WITH RELATIVES AND HEALTH PROFESSIONALS**	**MANAGING AND COPING WITH BD IN LATER LIFE**	Psychological help<br>Self-management<br>Medication<br>Distraction<br>Importance of a meaningful activity<br>Help from health professionals<br>Help from relative
**SUPPORT FROM THE THERAPIST**	**EXPERIENCE OF RECOVERY IN LATER LIFE**	Person's concept of recovery<br>Messages from health professionals about recovery

For peer review only

For peer review only - http://bmjopen.bmj.com/site/about/guidelines.xhtml

		Management strategies to achieve recovery<br><br>Gaining a meaningful activity to aid recovery<br><br>The importance of hope
**EXPERIENCE OF RECOVERY IN LATER LIFE**	**SEEKING HELP IN THE FUTURE**	Building relationship with therapist<br><br>Therapist transparency<br><br>Important goals for therapy<br><br>Building on strengths and resilience<br><br>Harder to seek help<br><br>Time running out to change
**ADAPTING RfCBT FOR OLDER PEOPLE**	**ADAPTING RfCBT FOR OLDER PEOPLE**	Session length<br><br>Memory and learning techniques<br><br>Enhancing the therapeutic experience<br><br>Using clear and simple language<br><br>Making study materials interesting and accessible<br><br>Booster sessions to optimise outcomes

Table 3: Themes

Theme 1: **Health and age-related changes in later life**

In general, the group appeared to have experienced a number of changes in later life consistent with the older adult literature. These included physical problems (e.g., arthritis, back problems and hearing difficulties), cognitive changes (e.g. decline in memory, concentration and increased distractibility) and behavioural changes (e.g. not playing sport or reading any longer). There was a

For peer review only

sense of frustration and sadness about not being able to engage in activities that they had previously enjoyed. What was interesting in this context, was how they made sense of these changes in relation to their BD diagnosis. With regards to their difficulties with memory and concentration, the group had trouble identifying whether this was part of the natural process of ageing or caused by their BD.

"Now how much of this is due to I'm getting older; how much is due to bipolar?" P002, FG2

This was particularly confusing as many people noticed differences in their memory depending on what mood state they were experiencing. So generally, when they felt low, they felt their memory difficulties worsened, however when their mood was higher some participants reported their memory problems appeared to dissipate.

"I am…on the ball. I can remember anything…As soon as my mood starts to dip then I start to not. I can't remember what I did the day before" P001, FG2"

The group reported changes in their family, work and social structure, meaning they had more time on their hands, which for some led to feelings of loneliness.

"I've lost such a lot of good friends…such a lot gone…I miss some of them around me" P004, FG1

Whilst changes in family, work and social structure are commonly reported by older adults (20), for this group having a life history of BD appeared to have had an additional impact. The group found having more time to themselves and dwelling upon events that had happened in the past (often related to situations that had arisen or been exacerbated by their behaviour whilst in a mood episode), led to feelings of guilt and shame. It was interesting that these stories were relayed with regrets that still felt quite raw, with people wishing they had behaved differently and treated family members with more respect.

"Coming to terms with events that have happened and you can't understand why that happened and why you did that….and with all the embarrassment to cope with" P001, FG1.

For peer review only - http://bmjopen.bmj.com/site/about/guidelines.xhtml

For other individuals in the group, not having to worry about the pressures of working life was seen in a more positive light with regards to the effect on their mood.

"I don't' have the stress of working life… and probably the stress makes it worse" P007, FG3

Theme 2: **Experience of BD in later life**

The group all reported a lifelong history of mood instability. In general, they felt their experience of both mania and depression had changed over the years, although patterns of change varied. What was common to all was a need to make sense of why this change was happening. Some people felt that they had fewer episodes now they were older, whereas others felt they had more but they were shorter and milder. In general, the group felt that they experienced more lows than high mood currently and some individuals felt their depressions had worsened over time compared to when they were younger. With regards to their depression, one participant stated:

"I have a knock down every day, two hours. Not heavy but I just close down" P002, FG1

In relation to mania, the majority of the group felt that their episodes were not as intense as they had been previously. The carer in the group described how their relative's mania was less physical and more cognitive than previously:

"It wasn't physical…It was more lots of disturbed thoughts" P006, FG1

The impact of living a life with a persistent mental health problem was evident within the group. Over the years they felt they had experienced stigma from society and a change in their social networks.

For peer review only

For peer review only - http://bmjopen.bmj.com/site/about/guidelines.xhtml

"And people think because you're mentally ill, in inverted commas, that you need to be treated differently" P005, FG3

Some members of the group felt they were now struggling with their sense of identity which had impacted on their confidence and self-esteem.

"I think it loses itself because you can't do the things you did… who am I now? What have I got to contribute?" P002, FG3

This struggle appeared to be linked to the combined consequences of the ageing process and the repeated episodes over the lifespan where the regrets still felt salient.

"I have to say my confidence is zilch now because of the events" P002, FG3

However not all members of the group were experiencing these problems: one individual described himself as having a very *"solid identity…with that workload"* (P007, FG3) which he attributed to the fact that he had a voluntary job. Another lady felt she had re-established her identity by going out and mixing with people and building up a community for herself:

"I'm a member of various organisations… I've got an activity every single day and I'm mixing with people and this helps enormously" (P005, FG3).

Here, the length of time since diagnosis does not appear to be a factor (P007 had a fairly recent time since diagnosis of 8 years and P005 received her diagnosis 26 years ago). Instead, having a role, a sense of purpose and belonging appears paramount to maintaining and enhancing a person's self-worth and resilience in later life.

***Theme 3:* Managing or coping with BD in later life**

The group had used a range of strategies over the years to manage their BD, including medication, psychological therapies, and self-management strategies. All of the group (apart from the relative) reported that they were taking medication and in general felt it had helped to stabilise their

For peer review only - http://bmjopen.bmj.com/site/about/guidelines.xhtml

episodes of BD. However, there were concerns about the effects of taking medication on their memory and concentration, which seemed to be intensifying as people were taking the medication over longer and longer periods of time.

Two members of the group had experience of their psychiatrist reducing their medication as they got older. They both felt that they had been over-medicated prior to the reduction.

"They saw I was a zombie and I didn't think I was capable of anything" (P005, FG1)

They both felt positive about the care that they had received from their psychiatrist in relation to the medication reduction.

The group acknowledged a change in approach over recent years with regards to psychological therapy and the majority of the group had some experience of psychological therapies (see table 1). A few members wondered about what other older friends with BD might think if offered a psychological approach.

"I'm not sure he would find that… a bit weird. A bit like whacky. Because he's nearly sixty, he's never been offered anything…." P001, FG1

Some members of the group felt they had learnt to manage their BD more effectively as they became older. Over the years, they felt they had learnt strategies to self-manage the condition through their own experience of living with BD and reading available information. They felt they were able to stabilise themselves more quickly in response to mood fluctuations, reporting conscious lifestyle changes including looking for triggers.

"I read up on it and I learnt all my trigger points; I now identify the illness whereas before I just thought it was the way my life was going" P005, FG1

For peer review only - http://bmjopen.bmj.com/site/about/guidelines.xhtml

For peer review only

One participant attributed this change in self-management to her CPN. The enhanced sense of responsibility to manage her own condition appeared a key factor in building the therapeutic relationship. This appeared to be significantly different to the care she had received in the past.

"Having a really good CPN who has given me responsibility, and believes in me, has really helped" (P005, FG3).

However, another participant felt that they were still engaged in a struggle with professionals which had persisted over the years. There was a sense of disappointment and frustration regarding the ongoing battle with professionals and an awareness that stigma related to living a life with BD may still be evident within some services.

"We're still fighting professionals who don't believe that we are capable of what we truly are" P005, FG1

The group used distraction as a way of coping on a more day to day basis, reporting activities such as gardening or voluntary jobs as a way of keeping the mind occupied and not focusing on past events. However, one group member acknowledged how distraction only worked as a strategy in the short-term.

"If you're doing something that doesn't require a lot of concentration, these things come back to the mind" P002, FG1

There appeared to be a mixture of experiences in relation to the help that individuals had received from relatives and friends to cope with their BD over the years. Maintaining a strong and supportive network of people around the person with BD was highlighted as an important coping factor by a number of group members, particularly as the person reaches their later years as roles change (e.g., retirement, family structure changes) and loneliness can become apparent.

Participants were able to identify times where they had found input from a relative/ friend helpful:

For peer review only - http://bmjopen.bmj.com/site/about/guidelines.xhtml

"if I went on a low my partner, my mate, my working partner knew the symptoms and he'd tell me, you know … you've got to be careful…" P004, FG1

However, one participant felt that her episodes were triggered by her husband's behaviour and now he was no longer in the home, her mood was stable and she in a stronger position to cope with her BD episodes.

"I no longer have that trigger so I am stable" P005, FG3

***Theme 4:* Experience of recovery in later life**

The concept of recovery in later life was introduced using the quote from the Scottish Recovery Network (see supplementary file 1). There appeared to be a division in the group with regards to their concept of 'recovery' in later life and their ability to recover with BD. Some participants questioned if the word recovery felt too final and a word like 'stability' may be more appropriate. These individuals were aligned with the traditional 'clinical' view of recovery and felt that being 'recovered' meant not experiencing any symptoms of BD in the future.

"Recovery means you're recovered…. You're cured and bipolar can't be cured…" P001, FG1

Recovering with BD felt like an unachievable aim:

"Something that I look for beyond the horizon" P002, FG1

In this sense, these participants questioned their ability to 'recover' in later life and to what degree they could move on and change. There appeared to be a link between their perception and messages they had received in the past about recovery.

"I've been told that you can never recover from bipolar" P002, FG1

For peer review only

For peer review only - http://bmjopen.bmj.com/site/about/guidelines.xhtml

337 However other members of the group felt they were 'recovered' or were in the process of
338 recovering. One individual described how recovery for the mental health team was the person not
339 going back into hospital, whereas for them it was getting back to work or engaging in a meaningful
340 activity. Therefore, he was able to differentiate between the messages from professionals regarding
341 'clinical recovery' and their own personal recovery journey.

342 Participants who identified with the more personal concept of recovery reported a range of
343 strategies to support their recovery journey; setting a goal and engaging in a meaningful activity felt
344 important, alongside the use of medication and self-management strategies. They acknowledged
345 how important hope was as part of their recovery journey.

346 *"You need hope" (P004, FG1)*

347 In general, there was this strong sense of wanting to contribute to society and look for new
348 opportunities following their lifetime of repeated episodes and transition into later life.

349 *"It's about setting out the right goals as well…isn't it?" (P004, FG1)*

350 Despite having to deal with the combined societal stigma of BD, and becoming older, some people
351 could clearly identify benefits from these simultaneous circumstances. There was a sense that they
352 had really 'survived' the struggle with living a life with bipolar and could use this wealth of
353 experience and knowledge to help their younger counterparts.

354 *"Our experiences in life have given us knowledge that we can help other people much better…we*
355 *know what works and what doesn't" (P005, FG1)*

356 Others found it harder to see these benefits, and seemed to have their own stigma regarding
357 becoming older and the limitations this puts on opportunities for personal growth and development:

358 *"I'm not sure at my age just to what degree I can move on" (P002, FG1).*

359 ***Theme 5:* Seeking help in the future**

For peer review only - http://bmjopen.bmj.com/site/about/guidelines.xhtml

Nearly all the members of the group had experience of psychological therapies in the past (see table 1). They were positive about the development of a tailored intervention specifically for their age group and they had some very clear ideas about the sort of relationship they would like with a potential therapist.

"They need to have listening skills. They need to be adapting body language and tone of voice and pitch of voice. They need to be emphatic…they need to be aware of the sort of problems we face…be able to get our trust" P005, FG3

They wanted to be treated with dignity and respect, but there was an additional need in this group for there to be a shared understanding of the wealth of experience the older person with BD was bringing to the therapeutic relationship.

They wanted transparency when it came to acknowledging any differences between the therapist and the client with regards to age, gender, ethnicity and the therapist's lack of BD diagnosis. They felt it was very important for the therapist to spend time building a relationship, allowing time at the beginning and the end of the session to really get to know the person and their living history. It was felt more time might be needed for this as their histories were often longer and more complex as a result of their age.

"It's about having that personal relationship…It takes two to three sessions to get the confidence" P004, FG2

The group wanted the therapist to work on issues such as assertiveness, confidence building and improving competence, maintaining an encouraging stance throughout. This seemed to be driven by a need for the therapeutic process to help challenge some of the stigmatising views of older adults in our society:

"We need loads of encouragement and appreciation that we are valued members of society" P005, FG1

For peer review only

In relation to seeking help, participants reflected on their own experiences and wondered if they would only engage in therapy when they were in an episode, perhaps influenced by negative experiences of care in the past. The carer in the group felt their relative found it harder to seek help now she was older because the repeated episodes over time (and limited life-span) had led to a greater sense of hopelessness.

Theme 6: **Adapting RfCBT for older people**

With regards to the specific recovery-focused intervention, the group appeared positive in general about the development of the specific therapy. They felt that the optimum session time was 50 minutes to an hour. They felt that it was necessary to make some adaptations to the therapy, based upon their experiences of living with BD in later life and the changes they had experienced. With regards to memory difficulties, they felt using strategies such as repetition and association would be helpful and writing summaries at the end of each session and revisiting these at the beginning of the next session.

"When you get older, you know, with sight problems and hearing problems you just need more resources… things written down… sort of more back up" P008, FG2

They suggested using images, film or audio recordings as a way of enhancing a person's therapeutic experience.

The group emphasised the importance of using clear and simple language and making study materials accessible and visually interesting.

"Some large sort of text…if it's complicated, it could be simplified" P006, FG2

For peer review only - http://bmjopen.bmj.com/site/about/guidelines.xhtml

The group were mixed in their opinion of how many sessions, some suggesting 6-8 sessions would be optimal and others thought up to a year was needed. The group suggested that booster sessions would be helpful at the end of therapy to re-visit strategies and enhance outcomes.

"If you've learnt some practical strategies…especially with CBT…they you've had some time to practice on your own" P008, FG2

DISCUSSION

The aim of this study was to better understand the experience of living BD, the concept of recovery and explore whether any adaptations were needed to the existing therapy RfCBT manual. The findings suggest that older people with BD are experiencing some of the same age-related issues as older people with other mental health issues. However, the consequence of having more time on their hands in later life (due to a reduction in social networks and loss of roles e.g. work) presented a specific challenge to this group of people. Time alone offered the opportunity to ruminate on negative events that had happened in the past which appear to be more salient in this group of people due to the nature of BD, raising issues such as guilt and shame. Interestingly, some participants felt a sense of stigma from mental health professionals and society in general which appeared to be enhanced by simultaneous condition of living with BD into older adulthood.

In general, the group reported an increase in lower mood which is consistent with previous research indicating older adults with BD present more predominantly with depressive symptoms (21). However, Laidlaw, Kishita and Chellingsworth (22) highlight that depression is not an automatic outcome of old age or an inevitable response to challenges of ageing. Therefore, developing an understanding for the predominance of depressive symptoms for older people with BD is important.

There were differences in the group's concept of recovery and whether the term was useful to describe their experience of living and coping with BD over the life-span, based on whether they saw this as a clinical or personal concept. A study exploring the concept of recovery in older people

For peer review only

For peer review only - http://bmjopen.bmj.com/site/about/guidelines.xhtml

versus younger people with mental health problems found that the older generation were not aiming towards a new or revised sense of identity (23). However, those who identified themselves as managing their difficulties competently felt they had sustained or recovered their sense of self. Findings from the current study support this as individuals who held positive views about their recovery journey and were engaging in meaningful activities, were the ones who felt their self-identity and confidence was now intact.

Whilst the group appeared positive about the development of an intervention for this population, one member questioned how other older people may perceive it. Research has found that older adults with depression do hold positive opinions about psychological therapy and if offered a choice would prefer them over psychotropic medication (however they may be less likely to be offered or receive this; 24)

The group had clear ideas about what they would like from a therapist and what adaptions they thought would be helpful for the RfCBT therapy, based upon their experience of living with BD in later life. These were consistent with guidelines developed for working with an older adult client group (22). There was a strong sense of the need to be valued and respected for their 'survival' of BD and the challenges which have presented throughout their lifespan. Knight and Laidlaw (25) identify 'wisdom' as a useful frame of reference when identifying and developing goals working with older depressed people. Therefore, building upon a person's 'wisdom' and life skills learnt in the face of adversity feels like an important part of the therapeutic process with this client group.

Considerations for clinical practice

The themes generated from the focus groups have led to a number of important recommendations for clinicians when delivering recovery focused CBT for older adults with BD. These are summarised in table 4 and map onto the 6 themes identified in the results section.

For peer review only - http://bmjopen.bmj.com/site/about/guidelines.xhtml

CONSIDERATIONS FOR DELIVERING RfCBT-OA
**Recovery stance**<br>• Explore the person's concept of recovery (traditional versus personal recovery). (4)<br>• Identify whether the word feels acceptable to use or find another to describe. (4)<br>• Identify and build upon any pre-existing strategies which have helped the person progress on their recovery journey. (4)<br>• Use 'hope' as a key message for helping a person progress towards their recovery goals. (4)<br>**Symptom management**<br>• Identify specific health and age-related changes which may be impacting upon a person's current presentation. (1)<br>• Explore whether there has been a change in symptoms over time (e.g. more depressive state now) and how they have coped with this. (2)<br>• Be aware that the consequence of repeated episodes over time (and limited life-span) may have led to a greater sense of hopelessness for change. (2)

For peer review only

- Allow time to explore issues such a guilt and shame in later life and the impact upon mood episodes now. (2)
- Explore the person's experience of receiving care from both relatives and health professionals and how this has affected their attitudes towards new opportunities for support. (3)

Specific adaptations

- Session length from 50 minutes to an hour. (6)
- To enhance memory and learning; use strategies such as repetition and association and write summaries at the end of each session (revisiting these at the beginning of the next session). (6)
- Use images, film or audio recordings as a way of enhancing a person's therapeutic experience. (6)
- Use clear and simple language and making study materials accessible and visually interesting. (6)
- Consider the use of booster sessions at the end of therapy to re-visit strategies and enhance outcomes. (6)

Therapist values

- Spend time building a relationship, exploring a longer and potentially more complex history, allowing time at the beginning and the end of the session. (5)
- Treat the older person with dignity and respect, develop a shared understanding of the wealth of experience the person with BD brings to the therapeutic relationship (challenging any perceived stigma the person may identify with related to living with BD as an older person). (5)
- Provide transparency and acknowledge any differences between the therapist and the client with regards to age, gender, ethnicity and the therapist's lack of BD diagnosis. (5)

For peer review only - http://bmjopen.bmj.com/site/about/guidelines.xhtml

Therapist focus

- Nurture pre-existing strengths and acknowledge the resilience already present of living a life with a long condition term. (5)
- Work on building assertiveness, confidence and competence to manage bipolar related experiences, maintaining an encouraging stance throughout. (5)

Table 4: Considerations for delivering RfCBT-OA

*1= Health and age-related changes, 2= experience of BD in later life, 3 = managing and coping with BD in later life, 4 = experience of recovery in later life, 5 = seeking help in the future, 6 = adapting RfCBT for older people).

Strengths and Limitations

The service user researcher co-facilitated the first group with the aim of facilitating open and honest discussions. They were able to identify with a lot of the issues raised by the group and their presence in the first group may have had some impact on some of the discussions.

When drawing any conclusions from the data it is evident that there are a number of methodological limitations which must be addressed. The participants taking part in the group are not representative of all older people with BD. They were a small, self-diagnosed group of people from the north-west of England who had an active interest in taking part in the study. They were all White British and all apart from one had (or had retired from) a professional working background.

Finally, the study employed a careful analysis to explore patterns in the individual's accounts of experience. However, all studies that are based upon self-report are constrained by the context and also subject to recall bias.

CONCLUSIONS

For peer review only - http://bmjopen.bmj.com/site/about/guidelines.xhtml

The study aimed to explore a number of topics relevant to living with BD in later life and use this information to enhance the pre-existing recovery focused therapy for older adults. In general, the group were positive about the development of a therapeutic approach for this specific cohort. They were able to give insight into the realities of living with BD across the life-span. The group highlighted a number of challenges that they had faced; however, nurturing the resilience and wisdom developed as a product of coping with adverse circumstances appears to be a key part of what they want from the therapeutic process.

Word count: 5196

DECLARATIONS

Ethics approval and consent to participate

The study was approved by the UK National Health Service (NHS) Ethics Committee process (REC ref: 15/NW/0330). All participants were asked to provide written consent prior to the focus groups.

Consent for publication

An unsigned copy of the consent form is available on request. All participants signed the consent form which provided consent to participate in the study and for their anonymised data, including quotations to be used for publication.

Availability of data and materials

The focus group(s) content and topics are available as a supplementary file 1. There are also additional quotes in supplementary file 2. The transcripts cannot be published or made available if requested to protect the anonymity of the participants who took part in the focus groups.

For peer review only - http://bmjopen.bmj.com/site/about/guidelines.xhtml

Competing interests

None declared.

Funding

This study was funded by the National Institute for Health Research through the Doctoral Research Fellowship programme. Grant No DRF-2014-07-094.

Author contributions

All authors contributed to development and preparation of the study design and topic guide. ET and RL conducted the focus groups. ET led the analysis, with contributions from FL and SJ. ET wrote the draft of the manuscript, which was proofed, edited and approved by FL, SJ and RL.

Acknowledgements

The authors would like to thank all of the individuals who took part in the study.

For peer review only

For peer review only - http://bmjopen.bmj.com/site/about/guidelines.xhtml

REFERENCES

1.Sajatovic, M., Gyulai, L., Calabrese, J.R., Thomas, R., Thompson, M.D., Wilson, B. et al. (2005) Maintenance treatment outcomes in older patients with bipolar I disorder. *Am J Geriatr Psychiatry, 13,* 305–311.

2. United Nations (2019): Population. https://www.un.org/en/sections/issues-depth/population/index.html (2019). Accessed 12 Oct 2020

3. Abdul-Hamid, W. K., Lewis-Cole, K., Holloway, F., & Silverman, A. M. (2015). Comparision of how old age psychiatry and general adult psychiatry services meet the needs of elderly people with functional mental illness: Cross-sectional survey. *The British Journal of Psychiatry: the Journal of Mental Science, 207(5),* 440-443.

4. National Institute for Health and Care Excellence (NICE). Bipolar disorder: assessment and management. 2014. https://www.nice.org.uk/guidance/cg185/chapter/1-Recommendations#managing-bipolar-depression-in-adults-in-secondary-care-2. Accessed 3 Dec 2020.

For peer review only

5. Hein, L., Dols, A., Eyler, L. T (2020) Bipolar disorders in older adults. In: Hantke, N., Etkin, A., O'Hara, R. Handbook of Mental Health and Aging (3rd Edition). Academic Press. Elsevier Inc, p 135-147.

6. Aziz, R., Lorberg, B., Tampi, R.R (2006) Treatments for late-life bipolar disorder. *Review Am J Geriatr Pharmacother 4(4):*347-64

7. Jones, S.H., Smith, G., Mulligan, L.D., Lobban, F., Law, H., Dunn, G., et al. (2015) Recovery-focused cognitive-behavioural therapy for recent-onset bipolar disorder: randomised controlled pilot trial. *Br J Psychiatry, 206*, 58–66.

8. Tyler, E., Lobban, F., Sutton, C., Depp, C., Johnson, S., Laidlaw, K. et al. (2016) Feasibility randomised controlled trial of Recovery-focused Cognitive Behavioural Therapy for Older Adults with bipolar disorder (RfCBT-OA): study protocol. BMJ Open. 6: e010590. doi:10.1136/bmjopen-2015-010590

9. Gould, R.L., Coulson, M.C., Howard, R.J. (2012a). Efficacy of Cognitive Behavioral Therapy for Anxiety Disorders in Older People: A Meta-Analysis and Meta-Regression of Randomized Controlled Trials. *J Am Geriatric Soc, 60* (2), 218-229.

10. Gould, R.L., Coulson, M.C., Howard, R.J. (2012b). Cognitive Behavioral Therapy for Depression in Older People: A Meta-Analysis and Meta-Regression of Randomized Controlled Trials. *J Am Geriatric Soc. 60* (10),1817-1830.

11. Campbell, M., Fitzpatrick, R., Haines, A., Kinmonth, A.L. (2000). Framework for design and evaluation of complex interventions to improve health. *Br Med J, 321,* 694–6.

12. Craig, P., Dieppe, P., Macintyre, S., Michie, S., Nazareth, I., Petticrew, M. (2008). Developing and evaluating complex interventions: the new Medical Research Council guidance. *Br Med J, 337,* 979–83.

For peer review only

13. Morgan, D. L. (1997). Focus groups as qualitative research (2nd ed.). Thousand Oaks, CA: Sage.

14. Bloor, M., Frankland, J., Thomas, M., & Robson, K. (2001). Focus groups in social research. Thousand Oaks, CA: Sage Publications Inc.

15. Baumgartner, T. A., Strong, C. H., Hensley, L. D. (2002). Conducting and reading research in health and human performance (3rd ed.). New York: McGraw-Hill.

16. Johnson, R. B., Christensen, L. B. (2004). Educational research: Quantitative, qualitative, and mixed approaches. Boston: Allyn and Bacon.

17. Ritchie, J., Spencer, L. (1994) Qualitative data analysis for applied policy research. In: Bryman A, Burgess RG. Analysing Qualitative Data. London: Routledge; p. 173-194.

18. Dixon-Woods, M. (2011). Using framework-based synthesis for conducting reviews of qualitative studies. *BMC Med, 9,* 39.

19. Furber, C. (2010). Framework analysis: a method for analysing qualitative data. *Afr J Midwifery Womens Health 4* (2), 97–100.

20. Age UK. (2010). Loneliness and Isolation Evidence Review. http://www.ageuk.org.uk/professional-resources-home/knowledge-hub-evidence statistics/evidence-reviews/. Accessed 5 Oct 2020.

21. Nivoli, A.M., Murru, A., Pacchiarotti, Valenti, M., Rosa, A., Hidalgo, Mazzei. D. et al. (2014). Bipolar disorder in the elderly: a cohort study comparing older and younger patients. *Acta Psychiatr Scand, 130,* 364–373.

22. Laidlaw, K., Kishita, N., Chellingsworth, M. (2016) A Clinicians guide to: CBT with older people. https://www.uea.ac.uk/documents/246046/18056409/CLINICIANS+GUIDE+TO+CBT+WITH+OLDER+PEOPLE+-+Beck-new.pdf/0adffe3a-2346-4fb2-b0b2-6bad3d20bd47 (2016). Accessed 3 Oct 2020

For peer review only - http://bmjopen.bmj.com/site/about/guidelines.xhtml

23. Daley S, Newton D, Slade M, Murray J, Banerjee S (2013). Development of a framework for recovery in older people with mental disorder. *Geriatric Psychiatry, 28,* (5), 522-529.

24. Gum, A.M., Areán, P.A., Hunkeler, E., Tang, L.M.A., Katon, W., Hitchcock, P. et al. (2006). Depression Treatment Preferences in Older Primary Care Patients. *Gerontologist, 46* (1), 14-22.

25. Knight, R.G., Laidlaw, K. (2009) Translational theory: A wisdom-based model for psychological interventions to enhance well-being in later life. In: Bengtson VL, Gans D, Putney NM, Silverstein M. (Eds.). Handbook of theories of aging (2nd ed). New York: Springer

For peer review only

For peer review only - http://bmjopen.bmj.com/site/about/guidelines.xhtml

Focus group topics and content

Focus group 1

1. Icebreaker

Focus group 1 started with an icebreaker (participants went around the room introducing themselves and telling the group an interesting fact about themselves).

2. Recovery approach introduced

The recovery approach was introduced and handed out on a piece of paper, defined as:

"Recovery is being able to live a meaningful and satisfying life, as defined by each person, in the presence or absence of symptoms. It is about having control over and input into your own life. Each individual's recovery, like his or her experience of the mental health problems or illness, is a unique and deeply personal process." (Scottish Recovery Network)

Participants were asked to comment on:

- their thoughts about this quote
- the term 'recovery'
- what recovery meant to them in later life

3. Experience of living with bipolar disorder in later life

Participants were asked to talk about their experience of living with bipolar disorder in later life.

4. Ways of coping with bipolar disorder in later life

Participants were asked about different ways of coping with the condition (including support from relatives and health professionals) in later life.

Focus group 2

1. Introductions

Focus group 2 started with everyone introducing themselves again, with the chance for new participants to tell an interesting fact about themselves.

2. Introducing recovery focused CBT and the stages of therapy

Recovery focused CBT therapy was introduced to the participants and the stages of therapy were discussed:

1. Introducing the recovery approach to clients;
2. Collection of information about current and historical mood and functioning;
3. Meaning and relevance of diagnosis;
4. Identification of recovery-informed therapy goals;
5. Initial formulation of relationships between mood experiences and progress towards recovery goals;

6. Identification and application of CBT techniques to address and facilitate positive coping;
7. Consideration of wider functioning issues in relation to recovery;
8. Development and completion of early warning signs (EWS) plan;
9. Development and completion of recovery plan;
10. Sharing lessons from therapy with key stakeholders.

Participants were asked to comment on:

- whether they thought the approach would be helpful with older adults
- what the strengths and weaknesses of the approach were
- what modifications (if any) would be needed for an older population

2. Session structure

Participants were told that the intervention would be delivered in one-to-one sessions and asked:

- what else might be important to include in therapy sessions for older people
- what might make therapy more accessible and effective for older people

3. Experience of therapy

Participants were asked to comment:

- if they had experience of receiving psychological therapy
- what they had found helpful/ unhelpful when accessing care and support (e.g. CPN, psychiatrist, therapist, relative) in the past
- what help they would want in the future

Focus group 3

1.Introductions

Focus group 3 started with everyone introducing themselves again.

2. Topics

All of the topics above were re-visited in focus group 3 so the three participants who hadn't attended 1 or 2 were able to share their ideas.

For peer review only

Theme	Example quotes
Health and age-related changes in later life	“My concentration isn’t very good and the sorts of books I read are different. I can’t read anything particularly frightening”<br>“I’m a bit slower but yeah, my memory is OK but like as soon as I get depressed, I get even worse”<br>“When my moods alright so it’s slightly elevated, I am … on the ball. I can remember anything…as soon as my mood starts to dip then I start to not…I can’t remember what I did the day before”<br>“I think my mood has an effect on my memory…it just gets hard as you get older”<br>“Just my ability to remember things. It’s in a limited way now. I put it down to the medication”<br>“I think the only thing I actually do is to listen to the radio and read a certain amount”<br>“We get fed up and we get frustrated and all those things because we can’t do what we did”<br>“As you get older there’s a lot more…quite often you’ve got more health issues you’ve got to deal with...those things can isolate XXX quite a lot. So, when she feels more isolated it can affect mood”<br>“Family members go and social situations change and health deteriorates”<br>“Family structure does change…it’s partly what upsets me, I suppose, when I’m in a low. You know, you look back...how many friends and how many family…have gone and you think Christ, you know, I’m the only one here. You don’t expect to be the last one.”<br>“Such a lot gone. And you just think I miss some of them around me and I must get what I want to do done”

For peer review only

	"The peer thing reduces when you get older... I think the thing that loses, if you get a good peer situation, your identity…when your older, there's a little bit of a problem trying to find your identity". "You're lonely and perhaps not as sharp as you were. So maybe it's a little bit more difficult…maybe we want it but how do we go about it. How do I go about getting involved back in life again"
Experience of BD in later life	"I found that with my manic episodes it's taken me less time to get stable again as the years have gone by. So whether that's an indication that in later life that they will be able to recover quicker and they're not as severe, I don't know." "It counts up to about eight whereas before it went up to nine" "I'm mostly downers. Well if I get in the middle of a picture I'm painting and I'm like enjoying it, I get a high because it's made me really happy…but most of it…I go quiet" "As the depressions have got worse everything pretty much shuts down and I'm depressed" "When I'm on a high I feel like my minds looking up…but when I'm down, I feel like my mind's closed" "I think it loses itself because you can't do the things you did…I do sometimes, and I'm sure everyone does, feels as if well, who am I now? What have I got to contribute?" "Launching yourself out was no problem when you're young, you know, because confidence" "You've got to believe in yourself and you have low self-esteem when you've got bipolar. And people think because you are mentally ill…you need you need to be treated differently"

For peer review only

	"I've got bipolar but I don't go telling other people because I know that, particularly my age group…if you're got to declare a mental illness, it's like well you're not one of them"<br>"It may be that...my psychological problems are sort of focused around get worse as they don't get solved or accepted all the time"<br>"Coming to terms with events that have happened, and you can't understand why that happened and why you did that and why you did this. And with all the embarrassment to cope with"<br>"Yeah, it's regret. Wishing that I'd been, behaved differently or treated somebody differently and that"<br>"If you're doing something that doesn't require a lot of concentration, these things come back to the mind"<br>"I think what's made a difference for my, for my mistakes is that I do have a living faith in God whom I can turn"<br>"What you've got to remember is that if you've got bipolar all your life you've got secrets…you've got personal secrets"<br>"And she kind of knows what she needs to do but there's a greater sense of hopelessness which might be just to do with less life span"
Managing and coping with BD in later life	"There's also been a change in, from my limited experience, that the medications seem to be only a, you know, pop a few pills.... that was the limit of it a while ago but there seems to be other areas being examined ...for example what you are doing"

For peer review only

	"I personally am terrified about what the medication – all the medication I'm on is going to do to me twenty years down the line... what effect it's going to have on my brain"<br>"As you grow older you don't need the amount of medication that you had when you were younger"<br>"Because of the medication being a lot more stable...Ive been able to undertake duties"<br>"I'm able to stabilise myself, sort of consciously change, lifestyle changes, depending on what mood I'm in... act on it quickly and so that I can reach a level ground again"<br>"I think it's a case of learning the illness and being able to manage the illness"<br>"I've painted a lot of pictures which is of course therapeutic"<br>"I think we are capable of doing an awful lot that people think we can't do and therefore we tend to play down our expectations and don't achieve our full potential...if people encourage us, it's surprising wheat we can achieve".<br>"It's about setting out the right goals as well...what older people might be capable of and talking about the sort of things that might be in our own heads individually and whether they are achievable".<br>"If you're having a good weeding session on the allotment, you're concentrating on what you're doing but if you're doing something that doesn't require a lot of concentration, these things come back to mind".<br>"I actually need to be doing at least two things to have a chance of distracting myself. So one way I get through the day when I'm low is to have the radio on but I'm also reading something"

For peer review only

	"I'm in charge of health and safety... and it, well it keeps me mind occupied doesn't it?<br><br>"I would expect health professionals to help you to bond to that community. I would expect them to know what is available to you, to signpost you"<br><br>"As you get older you get more assertive with your professionals"<br><br>"There is a difference now that the professionals treat me with respect and they ask my opinion and how often I want to see them"<br><br>"The younger doctors that I've had dealings with have been brilliant – they've been entirely professional but they've also been informal"<br><br>We're still fighting professionals who don't believe that we're capable of what we truly are and so we don't realise our full potential"<br><br>"A lot of older people they often had a partner that's helped kind of co-helped them co-manage the illness. And when that partner goes, so when my relative died... I didn't realise at the time but he helped modify"<br><br>"I've got a friend now...she phones me up in the morning at eight o'clock and says, how are you and we can be honest with one another"

For peer review only

Experience of recovery in later life	"This recovery we're just talking about... It's something that I look beyond the horizon"<br>"I think the word recovery... you aim for recovery...I think it should be called stability, not recovery"<br>"I don't think you ever recover...you manage it"<br>"Recovery for my therapy team was for me not to go back into hospital... But for me recovery was I really wanted to get another job. It was really important to me"<br>"I'm not sure at my age just to what degree I can move on really as time is flying by... I've been told you can never recover from bipolar"<br>"There is a great difficulty with the word recovery with professionals in the past by they are coming round"<br>"I'm a member of various organisations...I've got an activity every single day to do. And this helps... I'm out and about and I'm mixing with people and this helps enormously".<br>"I think It's possible to be recovered and I reckon that I am recovered now...I have the right medication...and I self-manage. I have a healthy lifestyle and I meet a lot of people"<br>"I've still got a life to live...I can meaningfully contribute to society"<br>"I want to move on from this. There's something better than this. If we haven't got hope, we're just going to wallow"<br>"You wouldn't want to recover if you didn't have hope"

For peer review only

Seeking help in the future	"I was thinking of having time round the therapy... having more time with an old person, sort of fifteen minutes before and fifteen minutes after... just being human together because it's hard doing therapy anyway"<br>"It's about having that personal relationship. It takes two or three sessions to get the confidence"<br>"I think as you get older, you perhaps become quicker at knowing whether you're going to click with somebody"<br>"And we're not as impressable"<br>"We can't be fooled too easily, can we?"<br>"They need to have listening skills. They need to be adapting body language and tone of voice and pitch of voice. They need to be empathic. And they need to be aware of the sort of problems that we face. And they need to get to the cause"<br>"You could raise the gender thing as an explicit issue... find out whether the client has any issues about the gender of the therapist"<br>"The therapist should try to make you feel assertive. Train you to be assertive"<br>"It's part of the therapist's duty to give you higher autonomy and assertiveness so you can fight your corner in a non-aggressive way but one that is assertive"<br>"Therapy focused partly on older age needs to help people to improve competence in whatever area they wanted"<br>"I think that could be a bit of a stumbling block for older people to be able to talk to younger people"

For peer review only

Adapting RfCBT for older people	"I think the idea of fifty minutes is partly that you can have a little bit of chant, you know, at the beginning. Sort of hello, how are you".<br>"When you get older, you know, with sight and problems and hearing problems you just need more resources...things written down... sort of more back up"<br>"She can't take lots of information in really quickly. So more time. Writing things down. Get her to write it down"<br>"Can you break it down...into simple clear language"<br>"Some large sort of text...if it's complicated it could be simplified"<br>"You need to use more images"<br>"It's more interesting to kind of ...engage with and it's easy to kind of, like you said about remembering things you have something to hang on to"<br>"I think a video would be extremely useful...then you can … a disk that you can put in computer and you could watch on the screen an instruction"<br>"If you have it once a fortnight, you've time to put into practice what's been said and what's been taught"<br>"It's quite useful to have a gap...you've learnt some practical strategies...then you've had time to practice on your own...without the weekly backup"<br>"The idea of a short-ish course and then maybe going back in"

For peer review only

Standards for Reporting Qualitative Research (SRQR)

http://www.equator-network.org/reporting-guidelines/srqr/

Title and abstract

	Page
**Title** - Concise description of the nature and topic of the study Identifying the study as qualitative or indicating the approach (e.g., ethnography, grounded theory) or data collection methods (e.g., interview, focus group) is recommended	1
**Abstract** - Summary of key elements of the study using the abstract format of the intended publication; typically includes background, purpose, methods, results, and conclusions	2

Introduction

**Problem formulation** - Description and significance of the problem/phenomenon studied; review of relevant theory and empirical work; problem statement	4
**Purpose or research question** - Purpose of the study and specific objectives or questions	4 & 5

Methods

**Qualitative approach and research paradigm** - Qualitative approach (e.g., ethnography, grounded theory, case study, phenomenology, narrative research) and guiding theory if appropriate; identifying the research paradigm (e.g., postpositivist, constructivist/ interpretivist) is also recommended; rationale**	5
**Researcher characteristics and reflexivity** - Researchers' characteristics that may influence the research, including personal attributes, qualifications/experience, relationship with participants, assumptions, and/or presuppositions; potential or actual interaction between researchers' characteristics and the research questions, approach, methods, results, and/or transferability	8
**Context** - Setting/site and salient contextual factors; rationale**	7
**Sampling strategy** - How and why research participants, documents, or events were selected; criteria for deciding when no further sampling was necessary (e.g., sampling saturation); rationale**	5 & 6
**Ethical issues pertaining to human subjects** - Documentation of approval by an appropriate ethics review board and participant consent, or explanation for lack thereof; other confidentiality and data security issues	5
**Data collection methods** - Types of data collected; details of data collection procedures including (as appropriate) start and stop dates of data collection and analysis, iterative process, triangulation of sources/methods, and modification of procedures in response to evolving study findings; rationale**	6, 7 & 8

For peer review only - http://bmjopen.bmj.com/site/about/guidelines.xhtml

For peer review only

**Data collection instruments and technologies** - Description of instruments (e.g., interview guides, questionnaires) and devices (e.g., audio recorders) used for data collection; if/how the instrument(s) changed over the course of the study	6 & 7
**Units of study** - Number and relevant characteristics of participants, documents, or events included in the study; level of participation (could be reported in results)	8 & 9
**Data processing** - Methods for processing data prior to and during analysis, including transcription, data entry, data management and security, verification of data integrity, data coding, and anonymization/de-identification of excerpts	7 & 8
**Data analysis** - Process by which inferences, themes, etc., were identified and developed, including the researchers involved in data analysis; usually references a specific paradigm or approach; rationale**	7 & 8
**Techniques to enhance trustworthiness** - Techniques to enhance trustworthiness and credibility of data analysis (e.g., member checking, audit trail, triangulation); rationale**	7 & 8

Results/findings

**Synthesis and interpretation** - Main findings (e.g., interpretations, inferences, and themes); might include development of a theory or model, or integration with prior research or theory	10 - 23
**Links to empirical data** - Evidence (e.g., quotes, field notes, text excerpts, photographs) to substantiate analytic findings	10 -23 & supplementary quote table

Discussion

**Integration with prior work, implications, transferability, and contribution(s) to the field -** Short summary of main findings; explanation of how findings and conclusions connect to, support, elaborate on, or challenge conclusions of earlier scholarship; discussion of scope of application/generalizability; identification of unique contribution(s) to scholarship in a discipline or field	21- 25
**Limitations** - Trustworthiness and limitations of findings	3 & 26

Other

**Conflicts of interest** - Potential sources of influence or perceived influence on study conduct and conclusions; how these were managed	27
**Funding** - Sources of funding and other support; role of funders in data collection, interpretation, and reporting	27

For peer review only - http://bmjopen.bmj.com/site/about/guidelines.xhtml

For peer review only

For peer review only - http://bmjopen.bmj.com/site/about/guidelines.xhtml

Developing a recovery-focused therapy for older people with bipolar disorder: A qualitative focus group study

Journal:	*BMJ Open*
Manuscript ID	bmjopen-2021-049829.R1
Article Type:	Original research
Date Submitted by the Author:	01-Jul-2021
Complete List of Authors:	Tyler, Elizabeth; Lancaster University, Lancaster University Division of Health Research<br>Lobban, Fiona; Lancaster University, Lancaster University Division of Health Research<br>Long, Rita; Lancaster University, Lancaster University Division of Health Research<br>Jones, Steven; Lancaster University, Lancaster University Division of Health Research
**Primary Subject Heading**:	Mental health
Secondary Subject Heading:	Health services research, Qualitative research, Geriatric medicine
Keywords:	MENTAL HEALTH, GERIATRIC MEDICINE, Depression & mood disorders < PSYCHIATRY

SCHOLARONE™
Manuscripts

I, the Submitting Author has the right to grant and does grant on behalf of all authors of the Work (as defined in the below author licence), an exclusive licence and/or a non-exclusive licence for contributions from authors who are: i) UK Crown employees; ii) where BMJ has agreed a CC-BY licence shall apply, and/or iii) in accordance with the terms applicable for US Federal Government officers or employees acting as part of their official duties; on a worldwide, perpetual, irrevocable, royalty-free basis to BMJ Publishing Group Ltd ("BMJ") its licensees and where the relevant Journal is co-owned by BMJ to the co-owners of the Journal, to publish the Work in this journal and any other BMJ products and to exploit all rights, as set out in our licence.

The Submitting Author accepts and understands that any supply made under these terms is made by BMJ to the Submitting Author unless you are acting as an employee on behalf of your employer or a postgraduate student of an affiliated institution which is paying any applicable article publishing charge ("APC") for Open Access articles. Where the Submitting Author wishes to make the Work available on an Open Access basis (and intends to pay the relevant APC), the terms of reuse of such Open Access shall be governed by a Creative Commons licence – details of these licences and which Creative Commons licence will apply to this Work are set out in our licence referred to above.

Other than as permitted in any relevant BMJ Author's Self Archiving Policies, I confirm this Work has not been accepted for publication elsewhere, is not being considered for publication elsewhere and does not duplicate material already published. I confirm all authors consent to publication of this Work and authorise the granting of this licence.

For peer review only

Developing a recovery-focused therapy for older people with bipolar disorder:

A qualitative focus group study

Dr Elizabeth Tyler : Lancaster University Division of Health Research, Lancaster, UK

Professor Fiona Lobban : Lancaster University Division of Health Research, Division of Health Research, Lancaster University, Lancaster, UK

Ms Rita Long: Lancaster University Division of Health Research, Lancaster University, Lancaster, UK

Professor Steven Jones : Lancaster University Division of Health Research, Lancaster University, Lancaster, UK

Corresponding author: Dr Elizabeth Tyler: e.tyler@lancaster.ac.uk

For peer review only - http://bmjopen.bmj.com/site/about/guidelines.xhtml

ABSTRACT

Objectives: As the world's population ages and there is increased awareness of Bipolar Disorder (BD) the number of older people living with the condition will rise substantially. There is no current evidence base for the effectiveness of psychological interventions for older adults with BD. This focus group study explored a number of topics to inform the development and delivery of a recovery-focused therapy (RfT) for older adults with BD.

Design: A qualitative focus group study.

Setting: Three focus groups were conducted at a University in the North-West of England.

Participants: Eight people took part in the focus groups; six older adults with BD, one carer and one friend.

Results: Participant's responses clustered into 6 themes: 1) health and age-related changes in later life, 2) the experience of BD in later life, 3) managing and coping with BD in later life, 4) recovery in later life, 5) seeking helping in the future 6) adapting RfT for older people.

Conclusions: Participants reported a range of health and age-related changes and strategies to manage their BD. Participants held mixed views about using the term 'recovery' in later life. Participants were in agreement that certain adaptations were needed for delivering RfT for older adults, based upon their experience of living with BD in later life. The data collected as part of the focus groups has led to a number of recommendations for delivering RfT for older adults with BD.

For peer review only

For peer review only - http://bmjopen.bmj.com/site/about/guidelines.xhtml

ARTICLE SUMMARY

Strengths and limitations of this study

To our knowledge this is the first study to involve older adults with BD to shape and develop a psychological intervention specifically for their cohort.

The study was designed and conducted in consultation with service user representation throughout, enhancing the quality, value and the relevance of the study.

The clinical recommendations for delivering RfT for older adults have been developed in partnership with individuals with lived experience of BD, carers and healthcare professionals.

The individuals with lived experience taking part in the group were not representative of all older people with BD. They were all White British and all had (or had retired from) a professional working background.

For peer review only

BACKGROUND

Approximately 25% of individuals living with Bipolar Disorder (BD) are age 60 or over (1). As the world's population ages (2) and awareness of the condition increases, the number of people living with BD into later life is expected to rise substantially. Research indicates that older adult specific services are better placed to meet the needs of those with mental health problems in later life, when compared to general adult services (3). The National Institute for Health and Care Excellence BD guideline (4) suggests that older adults with BD should be offered the same treatments as younger people, however there appear to be unique, clinical characteristics that feature in the older population (5), that may impact on their needs and response to treatment (6).

Older people with BD follow a chronic and persistent course (7), with recurrent mood episodes continuing beyond the age of 70 (8). The clinical features include poorer cognitive functioning, even during periods of mood stability (9), which may impact on functioning, leading to problems with finances, domestic roles, mobility and social and recreational activities (7). Rise et al's (10) systematic review found that older people with BD are more likely to present with conditions such as diabetes mellitus, cancer, thyroid disorder and hypertension compared to age matched controls. Older people with BD may be twice as likely to experiences stressful life events compared to healthy controls such as changes in familial structure, retirement, housing and finances (11), which can act as triggers for mood episodes (7). Studies have found that older people with BD are more likely to experience depressive episodes (12, 13) and there is some evidence to suggest milder episodes of mania, compared to their younger counterparts (14)

At present, there is no evidence base for the effectiveness of psychological interventions for older adults with BD. The aim of the study was to conduct a number of focus groups with people with lived experience of BD in later life, to adapt a recovery focused therapy (RfT) intervention developed for younger adults (15), so it could be offered to people over the age of 60. The focus group work sits within a larger study which consists of two phases (16).

For peer review only

For peer review only - http://bmjopen.bmj.com/site/about/guidelines.xhtml

The key aims of the focus groups were to: (1) explore the extent to which the original RfT intervention was acceptable to older adults with BD, (2) identify whether any adaptations would be needed to the existing manual and what support older adults would want from a therapist during therapy, (3) explore the experience of BD in later life; including the relationship with relatives and health professionals and the concept of recovery with BD.

METHOD

Design

The study was approved by the UK NHS Ethics Committee process (REC ref: 15/NW/0330). The British Medical Journal (BMJ) and the Medical Research Council (MRC) recommend the incorporation of qualitative research in the process of complex intervention development (17, 18). Focus group methodology (19) was chosen because the researcher was interested in understanding the topics from a diverse range of perspectives, moderating the discussion from a peripheral role (20). The Standards for Reporting Qualitative Research (SRQR) were adhered to and a copy of the checklist can be found in the supplementary information (Supplementary file 1).

Patient and public involvement

Patients and public involvement (PPI) representatives were involved in the study design process from the outset. The team has a service user advisory panel, led at the time by RL. The panel provided feedback on the original grant application and reviewed study documents, including participant information sheets, consent forms and topic guides. At the end of the focus group, participants were invited to remain in the study and form the service user reference group (SURG) to contribute towards the design, implementation and dissemination at phase two.

Sampling and recruitment

For peer review only

For peer review only - http://bmjopen.bmj.com/site/about/guidelines.xhtml

Participants were recruited via a confidential database of individuals who have previously consented to being approached about potential involvement in research studies. To be eligible to take part in the study, participants were required to identify themselves as a person over the age of 60 living with BD or be a relative or a friend of an older adult with BD (to offer a diverse range of perspectives), have the capacity to provide informed consent and have sufficient English language skills to read the information sheet and take part in the discussions.

We aimed to recruit approximately 6-12 participants, a combination of males and females, to take part in the focus groups, consistent with focus group methodology (19, 21, 22). We intended to recruit at least 6 people with lived experience of BD in later life and additional carers, relatives or friends to broaden the discussion. All interested individuals were invited to attend each of the 3 focus groups.

For peer review only

Topic guide for focus groups

A topic guide was developed with the research team (ET, SJ, FL and RL) and designed to loosely structure the focus group and lead the discussion. See supplementary file 2 for focus group topics and content. Different topics were explored in groups 1 and 2 (see table 1) and during group 3 the researcher re-visited all 6 topics to gather more rich and detailed information and ensure that all participants taking part had the opportunity to express their ideas on each of the areas. See table 2 for who attended each group.

Focus group	Topics explored
1	1. Overview of proposed therapy<br>2. Living with BD in later life<br>3. Experience of recovery in later life
2	4. Adapting RfT for older people

	5. What support people want from a therapist during therapy<br>6. Relationship with relatives and health professionals
3	1. ALL Topics re-visited

Table 1: Focus group topics

Procedure

The groups were conducted at a University in the North-West of England and lasted for approximately 90 minutes, consistent with focus group methodology (19). The first focus group was facilitated by two members of the research team (a service user researcher and the lead researcher). The service user researcher was not able attend groups 2 and 3 which were facilitated by the lead researcher alone. All groups were audio recorded, and transcribed prior to analysis. All participants provided written informed consent.

For peer review only

ANALYSIS

The focus groups were transcribed and analysed thematically using framework analysis (23), a popular way to analyse primary qualitative data in the area of healthcare (24). This allows for both deductive and inductive coding, with concepts or themes identified as coding categories *a priori* to be combined with other themes that emerge *de novo* (24). Five topics formed the original framework; living with BD in later life, experience of recovery in later life, adapting RfT for older people, what support people want from a therapist during therapy and the relationship with relatives and health professionals

Furber (25) identified five phases of framework analysis; familiarisation, a theoretical framework, indexing, charting and synthesis. The three transcripts were read and re-read by the lead researcher (ET) to aid the process of familiarisation, before undertaking the initial coding for transcript 1. Two members of the research team (SJ and FL) independently read and coded (indexed) transcript 1. A meeting took place within the team (ET, SJ and FL) to discuss the transcript, why the coded sections

For peer review only - http://bmjopen.bmj.com/site/about/guidelines.xhtml

had been interpreted as meaningful and to discuss new codes and the development of the theoretical framework. The new framework was then applied to transcripts 2 and 3. Team meetings took place to discuss any further amendments to the framework, based on the emergence of new codes. The final framework was a representative of the entire data set collected from the 3 focus groups.

Reflexivity

The researcher was aware that they (and other members of the research team) were approaching the study with a set of pre-conceived ideas about the recovery focused approach and the direction of therapy. SJ developed the original recovery focused CBT manual for an adult BD population. SJ and FL were involved in a recently completed a trial exploring its feasibility and acceptability (8). Subsequently ET, SJ and FL were involved in a case series looking at the application of this approach for individuals with a more established BD diagnosis. The researcher aimed to facilitate the discussion regarding these topics in an open fashion to ensure that the participants felt able to give open and honest feedback about the approach. Similarly, when analysing and interpreting the results, the role of potential bias was highlighted to ensure that the results were a true reflection of the participant's ideas, rather than the team selecting the responses which aligned with their personal view on the topic.

RESULTS

Participants

All participants were invited to attended each focus group; however, this was not achievable due to their various commitments and therefore we were flexible in response to their availability. As shown in table 2, eight participants took part across the 3 focus groups attending 1-3 groups each; 6 service users with lived experience and 1 carer and 1 friend (who also had a diagnosis of BD and identified herself as moving into later life).

For peer review only

Participant number	P001	P002	P003	P004	P005	P006	P007	P008
**Focus group attended**	1,2	1,2,3	1	1,2	1,3	1,2	2,3	2
**Service user/ carer/ friend**	Friend	Service user	Service user	Service user	Service user	Carer	Service user	Service user

Table 2: Groups attended

For peer review only

Participant characteristic	N (6)
**Ethnicity**	
White British	6
**Age**	
65- 70	2
70-75	2
75-80	2
**Gender**	
Male	3
Female	3
**Length of Diagnosis**	
Less than 10 years	1
10-15 years	1
15-20 years	1
20-30 years	2
30+ years	1
**Current occupation**	
Retired	5
Director of a company	1
**Occupation prior to retirement**	
Director of a company	1
Company secretary	1
Teacher	1
University researcher	1
Nursing	1
**Past experience of psychological therapy**	
Yes	4
No	2
**Type of therapy**	
CBT	3
Mindfulness	1

Table 3: Participant characteristic for individuals with lived experience

Participants with lived experience ages ranged from 67 to 77 years (M = 72), with an established diagnosis, ranging from 8 to 38 years (M = 20).

For peer review only - http://bmjopen.bmj.com/site/about/guidelines.xhtml

PRELIMINARY THEME	FINAL THEME	SUB-THEME
**LIVING WITH BD IN LATER LIFE**	**HEALTH AND AGE-RELATED CHANGES IN LATER LIFE**	Cognitive symptoms<br>Behavioural changes<br>Physical symptoms<br>Social network changes<br>Loneliness and isolation
**LIVING WITH BD IN LATER LIFE**	**EXPERIENCE OF BD IN LATER LIFE**	Change in psychological symptoms<br>Identity<br>Stigma<br>Guilt and shame
**LIVING WITH BD IN LATER LIFE**<br>**RELATIONSHIP WITH RELATIVES AND HEALTH PROFESSIONALS**	**MANAGING AND COPING WITH BD IN LATER LIFE**	Psychological help<br>Self-management<br>Medication<br>Distraction<br>Importance of a meaningful activity<br>Help from health professionals<br>Help from relative
**EXPERIENCE OF RECOVERY IN LATER LIFE**	**EXPERIENCE OF RECOVERY IN LATER LIFE**	Person's concept of recovery<br>Messages from health professionals about recovery<br>Management strategies to achieve recovery<br>Gaining a meaningful activity to aid recovery<br>The importance of hope

For peer review only - http://bmjopen.bmj.com/site/about/guidelines.xhtml

**SUPPORT FROM THE THERAPIST**	**SEEKING HELP IN THE FUTURE**	Building relationship with therapist<br>Therapist transparency<br>Important goals for therapy<br>Building on strengths and resilience<br>Harder to seek help<br>Time running out to change
**ADAPTING RfT FOR OLDER PEOPLE**	**ADAPTING RfT FOR OLDER PEOPLE**	Session length<br>Memory and learning techniques<br>Enhancing the therapeutic experience<br>Using clear and simple language<br>Making study materials interesting and accessible<br>Booster sessions to optimise outcomes

Table 4: Themes

Themes

There was a great deal of rich data regarding how participants described perceived changes during their later years. Once the team met to code focus group 1, using a combination of both inductive and deductive analysis, they agreed that the framework should be revised to reflect the data. The original topic "living with BD in later life" was broadened to encompass 3 themes; "health and age-related changes in later life", "experience of BD in later life", "managing and coping with BD in later life", following an inductive approach. Initial topics of interest "relationship with relatives and health professionals" became subthemes within the new theme "managing and coping with bipolar in later life". The original topic "what support people want from a therapist" became a new theme "seeking helping in the future". The original topics of interest "experience of recovery in later life" and "adapting RfT for older people" became the final themes, following a deductive approach. Please see

For peer review only

For peer review only - http://bmjopen.bmj.com/site/about/guidelines.xhtml

188 table 4 for the preliminary and final themes. Additional example quotes can be found in
189 supplementary file 3.

190 ***Theme 1:* Health and age-related changes in later life**

191 In general, the group appeared to have experienced a number of changes in later life. These included
192 physical problems (e.g., arthritis, back problems and hearing difficulties), cognitive changes (e.g.
193 decline in memory, concentration and increased distractibility) and behavioural changes (e.g. not
194 playing sport or reading any longer). There was a sense of frustration and sadness about not being
195 able to engage in activities that they had previously enjoyed. What was interesting in this context,
196 was how they made sense of these changes in relation to their BD diagnosis. With regards to their
197 difficulties with memory and concentration, the group had trouble identifying whether this was part
198 of the natural process of ageing or caused by their BD.

199 *"Now how much of this is due to I'm getting older; how much is due to bipolar?" P002, FG2*

200 This was particularly confusing as many people noticed differences in their memory depending on
201 what mood state they were experiencing. So generally, when they felt low, they felt their memory
202 difficulties worsened, however when their mood was higher some participants reported their
203 memory problems appeared to dissipate.

204 *"I am…on the ball. I can remember anything…As soon as my mood starts to dip then I start to not. I*
205 *can't remember what I did the day before" P001, FG2"*

206 The group reported changes in their family, work and social structure, meaning they had more time
207 on their hands, which for some led to feelings of loneliness.

208 *"I've lost such a lot of good friends…such a lot gone…I miss some of them around me" P004, FG1*

209 Whilst changes in family, work and social structure are commonly reported by older adults (20), for
210 this group having a life history of BD appeared to have had an additional impact. The group found

For peer review only - http://bmjopen.bmj.com/site/about/guidelines.xhtml

having more time to themselves and dwelling upon events that had happened in the past (often related to situations that had arisen or been exacerbated by their behaviour whilst in a mood episode), led to feelings of guilt and shame. It was interesting that these stories were relayed with regrets that still felt quite raw, with people wishing they had behaved differently and treated family members with more respect.

"Coming to terms with events that have happened and you can't understand why that happened and why you did that….and with all the embarrassment to cope with" P001, FG1.

For other individuals in the group, not having to worry about the pressures of working life was seen in a more positive light with regards to the effect on their mood.

"I don't have the stress of working life… and probably the stress makes it worse" P007, FG3

***Theme 2:* Experience of BD in later life**

The group all reported a lifelong history of mood instability. In general, they felt their experience of both mania and depression had changed over the years, although patterns of change varied. What was common to all was a need to make sense of why this change was happening. Some people felt that they had fewer episodes now they were older, whereas others felt they had more but they were shorter and milder. In general, the group felt that they experienced more lows than high mood currently and some individuals felt their depressions had worsened over time compared to when they were younger. With regards to their depression, one participant stated:

"I have a knock down every day, two hours. Not heavy but I just close down" P002, FG1

In relation to mania, the majority of the group felt that their episodes were not as intense as they had been previously. The carer in the group described how their relative's mania was less physical and more cognitive than previously:

"It wasn't physical…It was more lots of disturbed thoughts" P006, FG1

For peer review only - http://bmjopen.bmj.com/site/about/guidelines.xhtml

The impact of living a life with a persistent mental health problem was evident within the group. Over the years they felt they had experienced stigma from society and a change in their social networks.

“And people think because you’re mentally ill, in inverted commas, that you need to be treated differently” P005, FG3

Some members of the group felt they were now struggling with their sense of identity which had impacted on their confidence and self-esteem.

“I think it loses itself because you can’t do the things you did… who am I now? What have I got to contribute?” P002, FG3

This struggle appeared to be linked to the combined consequences of the ageing process and the repeated episodes over the lifespan where the regrets still felt salient.

“I have to say my confidence is zilch now because of the events” P002, FG3

However not all members of the group were experiencing these problems: one individual described himself as having a very *“solid identity…with that workload”* (P007, FG3) which he attributed to the fact that he had a voluntary job. Another lady felt she had re-established her identity by going out and mixing with people and building up a community for herself:

“I’m a member of various organisations… I’ve got an activity every single day and I’m mixing with people and this helps enormously” (P005, FG3).

Here, the length of time since diagnosis does not appear to be a factor (P007 had a fairly recent time since diagnosis of 8 years and P005 received her diagnosis 26 years ago). Instead, having a role, a sense of purpose and belonging appears paramount to maintaining and enhancing a person’s self-worth and resilience in later life.

For peer review only - http://bmjopen.bmj.com/site/about/guidelines.xhtml

***Theme 3:* Managing or coping with BD in later life**

The group had used a range of strategies over the years to manage their BD, including medication, psychological therapies, and self-management strategies. All of the group (apart from the relative) reported that they were taking medication and in general felt it had helped to stabilise their episodes of BD. However, there were concerns about the effects of taking medication on their memory and concentration, which seemed to be intensifying as people were taking the medication over longer and longer periods of time.

Two members of the group had experience of their psychiatrist reducing their medication as they got older. They both felt that they had been over-medicated prior to the reduction.

"They saw I was a zombie and I didn't think I was capable of anything" (P005, FG1)

They both felt positive about the care that they had received from their psychiatrist in relation to the medication reduction.

The group acknowledged a change in approach over recent years with regards to psychological therapy and the majority of the group had some experience of psychological therapies (see table 1). A few members wondered about what other older friends with BD might think if offered a psychological approach.

"I'm not sure he would find that… a bit weird. A bit like whacky. Because he's nearly sixty, he's never been offered anything…." P001, FG1

Some members of the group felt they had learnt to manage their BD more effectively as they became older. Over the years, they felt they had learnt strategies to self-manage the condition through their own experience of living with BD and reading available information. They felt they were able to stabilise themselves more quickly in response to mood fluctuations, reporting conscious lifestyle changes including looking for triggers.

For peer review only - http://bmjopen.bmj.com/site/about/guidelines.xhtml

"I read up on it and I learnt all my trigger points; I now identify the illness whereas before I just thought it was the way my life was going" P005, FG1

One participant attributed this change in self-management to her CPN. The enhanced sense of responsibility to manage her own condition appeared a key factor in building the therapeutic relationship. This appeared to be significantly different to the care she had received in the past.

"Having a really good CPN who has given me responsibility, and believes in me, has really helped" (P005, FG3).

However, another participant felt that they were still engaged in a struggle with professionals which had persisted over the years. There was a sense of disappointment and frustration regarding the ongoing battle with professionals and an awareness that stigma related to living a life with BD may still be evident within some services.

"We're still fighting professionals who don't believe that we are capable of what we truly are" P005, FG1

The group used distraction as a way of coping on a more day to day basis, reporting activities such as gardening or voluntary jobs as a way of keeping the mind occupied and not focusing on past events. However, one group member acknowledged how distraction only worked as a strategy in the short-term.

"If you're doing something that doesn't require a lot of concentration, these things come back to the mind" P002, FG1

There appeared to be a mixture of experiences in relation to the help that individuals had received from relatives and friends to cope with their BD over the years. Maintaining a strong and supportive network of people around the person with BD was highlighted as an important coping factor by a

For peer review only

For peer review only - http://bmjopen.bmj.com/site/about/guidelines.xhtml

number of group members, particularly as the person reaches their later years as roles change (e.g., retirement, family structure changes) and loneliness can become apparent.

Participants were able to identify times where they had found input from a relative/ friend helpful:

“if I went on a low my partner, my mate, my working partner knew the symptoms and he’d tell me, you know … you’ve got to be careful…” P004, FG1

However, one participant felt that her episodes were triggered by her husband’s behaviour and now he was no longer in the home, her mood was stable and she in a stronger position to cope with her BD episodes.

“I no longer have that trigger so I am stable” P005, FG3

***Theme 4:* Experience of recovery in later life**

The concept of recovery in later life was introduced using the quote from the Scottish Recovery Network (see supplementary file 1). There appeared to be a division in the group with regards to their concept of ‘recovery’ in later life and their ability to recover with BD. Some participants questioned if the word recovery felt too final and a word like ‘stability’ may be more appropriate. These individuals were aligned with the traditional ‘clinical’ view of recovery and felt that being ‘recovered’ meant not experiencing any symptoms of BD in the future.

“Recovery means you’re recovered…. You’re cured and bipolar can’t be cured…” P001, FG1

Recovering with BD felt like an unachievable aim:

“Something that I look for beyond the horizon” P002, FG1

In this sense, these participants questioned their ability to ‘recover’ in later life and to what degree they could move on and change. There appeared to be a link between their perception and messages they had received in the past about recovery.

For peer review only

For peer review only - http://bmjopen.bmj.com/site/about/guidelines.xhtml

"I've been told that you can never recover from bipolar" P002, FG1

However other members of the group felt they were 'recovered' or were in the process of recovering. One individual described how recovery for the mental health team was the person not going back into hospital, whereas for them it was getting back to work or engaging in a meaningful activity. Therefore, he was able to differentiate between the messages from professionals regarding 'clinical recovery' and their own personal recovery journey.

Participants who identified with the more personal concept of recovery reported a range of strategies to support their recovery journey; setting a goal and engaging in a meaningful activity felt important, alongside the use of medication and self-management strategies. They acknowledged how important hope was as part of their recovery journey.

"You need hope" (P004, FG1)

In general, there was this strong sense of wanting to contribute to society and look for new opportunities following their lifetime of repeated episodes and transition into later life.

"It's about setting out the right goals as well…isn't it?" (P004, FG1)

Despite having to deal with the combined societal stigma of BD, and becoming older, some people could clearly identify benefits from these simultaneous circumstances. There was a sense that they had really 'survived' the struggle with living a life with bipolar and could use this wealth of experience and knowledge to help their younger counterparts.

"Our experiences in life have given us knowledge that we can help other people much better…we know what works and what doesn't" (P005, FG1)

Others found it harder to see these benefits, and seemed to have their own stigma regarding becoming older and the limitations this puts on opportunities for personal growth and development:

"I'm not sure at my age just to what degree I can move on" (P002, FG1).

For peer review only

***Theme 5:* Seeking help in the future**

Nearly all the members of the group had experience of psychological therapies in the past (see table 1). They were positive about the development of a tailored intervention specifically for their age group and they had some very clear ideas about the sort of relationship they would like with a potential therapist.

"They need to have listening skills. They need to be adapting body language and tone of voice and pitch of voice. They need to be emphatic…they need to be aware of the sort of problems we face…be able to get our trust" P005, FG3

They wanted to be treated with dignity and respect, but there was an additional need in this group for there to be a shared understanding of the wealth of experience the older person with BD was bringing to the therapeutic relationship.

They wanted transparency when it came to acknowledging any differences between the therapist and the client with regards to age, gender, ethnicity and the therapist's lack of BD diagnosis. They felt it was very important for the therapist to spend time building a relationship, allowing time at the beginning and the end of the session to really get to know the person and their living history. It was felt more time might be needed for this as their histories were often longer and more complex as a result of their age.

"It's about having that personal relationship…It takes two to three sessions to get the confidence" P004, FG2

The group wanted the therapist to work on issues such as assertiveness, confidence building and improving competence, maintaining an encouraging stance throughout. This seemed to be driven by a need for the therapeutic process to help challenge some of the stigmatising views of older adults in our society:

For peer review only

For peer review only - http://bmjopen.bmj.com/site/about/guidelines.xhtml

"We need loads of encouragement and appreciation that we are valued members of society" P005, FG1

In relation to seeking help, participants reflected on their own experiences and wondered if they would only engage in therapy when they were in an episode, perhaps influenced by negative experiences of care in the past. The carer in the group felt their relative found it harder to seek help now she was older because the repeated episodes over time (and limited life-span) had led to a greater sense of hopelessness.

***Theme 6:* Adapting RfT for older people**

With regards to the specific recovery-focused intervention, the group appeared positive in general about the development of the specific therapy. They felt that the optimum session time was 50 minutes to an hour. They felt that it was necessary to make some adaptations to the therapy, based upon their experiences of living with BD in later life and the changes they had experienced. With regards to memory difficulties, they felt using strategies such as repetition and association would be helpful and writing summaries at the end of each session and revisiting these at the beginning of the next session.

"When you get older, you know, with sight problems and hearing problems you just need more resources… things written down… sort of more back up" P008, FG2

They suggested using images, film or audio recordings as a way of enhancing a person's therapeutic experience.

The group emphasised the importance of using clear and simple language and making study materials accessible and visually interesting.

"Some large sort of text…if it's complicated, it could be simplified" P006, FG2

For peer review only

For peer review only - http://bmjopen.bmj.com/site/about/guidelines.xhtml

The group were mixed in their opinion of how many sessions, some suggesting 6-8 sessions would be optimal and others thought up to a year was needed. The group suggested that booster sessions would be helpful at the end of therapy to re-visit strategies and enhance outcomes.

“If you’ve learnt some practical strategies…especially with CBT…they you’ve had some time to practice on your own” P008, FG2

DISCUSSION

The aim of this study was to better understand the experience of living BD, the concept of recovery and explore whether any adaptations were needed to the existing therapy RfT manual. The findings suggest that the group were experiencing similar health and age-related changes compared to other older people living with BD, such as changes in cognition (26) and an increase in physical health conditions (27, 28). The group also appeared to be experiencing the same issues as older people with other mental health issues such as losses, loneliness and isolation (29). However, the consequence of having more time on their hands in later life (due to a reduction in social networks and loss of roles (e.g. work) presented a specific challenge to this group of people. Time alone offered the opportunity to ruminate on negative events that had happened in the past which appear to be more salient in this group of people due to the nature of BD, raising issues such as guilt and shame. Interestingly, some participants felt a sense of stigma from mental health professionals and society in general which appeared to be enhanced by simultaneous condition of living with BD into older adulthood.

In general, the group reported an increase in lower mood which is consistent with previous research indicating older adults with BD present more predominantly with depressive symptoms (12, 13) and milder episodes of mania, consistent with previous research (14). Laidlaw, Kishita and Chellingsworth (30) highlight that depression is not an automatic outcome of old age or an inevitable

For peer review only

response to challenges of ageing. Therefore, developing an understanding for the predominance of depressive symptoms for older people with BD is important.

There were differences in the group's concept of recovery and whether the term was useful to describe their experience of living and coping with BD over the life-span, based on whether they saw this as a clinical or personal concept. A study exploring the concept of recovery in older people versus younger people with mental health problems found that the older generation were not aiming towards a new or revised sense of identity (31). However, those who identified themselves as managing their difficulties competently felt they had sustained or recovered their sense of self. Findings from the current study support this as individuals who held positive views about their recovery journey and were engaging in meaningful activities, were the ones who felt their self-identity and confidence was now intact.

Whilst the group appeared positive about the development of an intervention for this population, one member questioned how other older people may perceive it. Research has found that older adults with depression do hold positive opinions about psychological therapy and if offered a choice would prefer them over psychotropic medication (however they may be less likely to be offered or receive this; 32)

Considerations for clinical practice

The themes generated from the focus groups have led to a number of important recommendations for clinicians when delivering recovery focused therapy for older people with BD. There were consistencies with younger individuals' priorities for recovery, where hope has been highlighted as a key part of the recovery process (33). The group had clear ideas about what they would like from a therapist and wanted to work on areas of personal growth such as building assertiveness, confidence and competence. These targets identified with older people align with findings from Jones et al's (15) RfT trial with a younger cohort where improvements in personal recovery were associated with

For peer review only - http://bmjopen.bmj.com/site/about/guidelines.xhtml

improvements in personal growth and self-esteem (34). The group identified a number of adaptions they thought would be helpful for RfT for older people, based upon their experience of living with BD in later life. These were consistent with guidelines developed for working with an older adult client group (30). There was a strong sense of the need to be valued and respected for their 'survival' of BD and the challenges which have presented throughout their lifespan. Knight and Laidlaw (35) identify 'wisdom' as a useful frame of reference when identifying and developing goals working with older depressed people. Therefore, building upon a person's 'wisdom' and life skills learnt in the face of adversity feels like an important part of the therapeutic process with this client group.

This is the first study to involve older people with BD to shape and develop an intervention for their cohort, enhancing the quality, value and relevance of the recommendations. The recommendations have been used in the second phase of the programme of work which has evaluated RfT for older people in a randomised controlled trial (16). They also provide a helpful framework for clinicians working with older people with BD in wider clinical practice, based upon the service user's priorities. The recommendations are summarised in table 5 and map onto the 6 themes identified in the results section.

For peer review only - http://bmjopen.bmj.com/site/about/guidelines.xhtml

CONSIDERATIONS FOR DELIVERING RfT for older people

Recovery stance

- Explore the person's concept of recovery (traditional versus personal recovery). (4)
- Identify whether the word feels acceptable to use or find another to describe. (4)
- Identify and build upon any pre-existing strategies which have helped the person progress on their recovery journey. (4)
- Use 'hope' as a key message for helping a person progress towards their recovery goals. (4)

Symptom management

- Identify specific health and age-related changes which may be impacting upon a person's current presentation. (1)
- Explore whether there has been a change in symptoms over time (e.g. more depressive state now) and how they have coped with this. (2)
- Be aware that the consequence of repeated episodes over time (and limited life-span) may have led to a greater sense of hopelessness for change. (2)
- Allow time to explore issues such a guilt and shame in later life and the impact upon mood episodes now. (2)
- Explore the person's experience of receiving care from both relatives and health professionals and how this has affected their attitudes towards new opportunities for support. (3)

Specific adaptations

- Session length from 50 minutes to an hour. (6)
- To enhance memory and learning; use strategies such as repetition and association and write summaries at the end of each session (revisiting these at the beginning of the next session). (6)

For peer review only

- Use images, film or audio recordings as a way of enhancing a person's therapeutic experience. (6)
- Use clear and simple language and making study materials accessible and visually interesting. (6)
- Consider the use of booster sessions at the end of therapy to re-visit strategies and enhance outcomes. (6)

Therapist values

- Spend time building a relationship, exploring a longer and potentially more complex history, allowing time at the beginning and the end of the session. (5)
- Treat the older person with dignity and respect, develop a shared understanding of the wealth of experience the person with BD brings to the therapeutic relationship (challenging any perceived stigma the person may identify with related to living with BD as an older person). (5)
- Provide transparency and acknowledge any differences between the therapist and the client with regards to age, gender, ethnicity and the therapist's lack of BD diagnosis. (5)

Therapist focus

- Nurture pre-existing strengths and acknowledge the resilience already present of living a life with a long condition term. (5)
- Work on building assertiveness, confidence and competence to manage bipolar related experiences, maintaining an encouraging stance throughout. (5)

Table 5: Considerations for delivering RfT for older people

*1= Health and age-related changes, 2= experience of BD in later life, 3 = managing and coping with BD in later life, 4 = experience of recovery in later life, 5 = seeking help in the future, 6 = adapting RfT for older people).

For peer review only

For peer review only - http://bmjopen.bmj.com/site/about/guidelines.xhtml

468 ***Strengths and Limitations***

469 The service user researcher co-facilitated the first group with the aim of facilitating open and honest 470 discussions. They were able to identify with a lot of the issues raised by the group and their presence 471 in the first group may have had some impact on some of the discussions.

472 When drawing any conclusions from the data it is evident that there are a number of methodological 473 limitations which must be addressed. The individuals with lived experience taking part in the group 474 are not representative of all older people with BD. They were a small, self-diagnosed group of people 475 from the north-west of England who had an active interest in taking part in the study. They were all 476 White British and all had (or had retired from) a professional working background.

477 Finally, the study employed a careful analysis to explore patterns in the individual's accounts of 478 experience. However, all studies that are based upon self-report are constrained by the context and 479 also subject to recall bias.

480 **CONCLUSIONS**

481 The study aimed to explore a number of topics relevant to living with BD in later life and use this 482 information to enhance the pre-existing recovery focused therapy for older adults. In general, the 483 group were positive about the development of a therapeutic approach for this specific cohort. They 484 were able to give insight into the realities of living with BD across the life-span. The group 485 highlighted a number of challenges that they had faced; however, nurturing the resilience and 486 wisdom developed as a product of coping with adverse circumstances appears to be a key part of 487 what they want from the therapeutic process.

488 **Word count**: 5479

489

490

For peer review only - http://bmjopen.bmj.com/site/about/guidelines.xhtml

DECLARATIONS

Ethics approval and consent to participate

The study was approved by the UK National Health Service (NHS) Ethics Committee process (REC ref: 15/NW/0330). All participants were asked to provide written consent prior to the focus groups.

Consent for publication

An unsigned copy of the consent form is available on request. All participants signed the consent form which provided consent to participate in the study and for their anonymised data, including quotations to be used for publication.

Availability of data and materials

The focus group(s) content and topics are available as a supplementary file 1. There are also additional quotes in supplementary file 2. The transcripts cannot be published or made available if requested to protect the anonymity of the participants who took part in the focus groups.

Competing interests

None declared.

Funding

This study was funded by the National Institute for Health Research through the Doctoral Research Fellowship programme. Grant No DRF-2014-07-094.

Author contributions

All authors contributed to development and preparation of the study design and topic guide. ET and RL conducted the focus groups. ET led the analysis, with contributions from FL and SJ. ET wrote the draft of the manuscript, which was proofed, edited and approved by FL, SJ and RL.

For peer review only

512 **Acknowledgements**

513 The authors would like to thank all of the individuals who took part in the study.

514

515

516

517

518

519

520

521

522

523

524

525

526

527

528

529

530

For peer review only

For peer review only - http://bmjopen.bmj.com/site/about/guidelines.xhtml

REFERENCES

1. Sajatovic, M., Gyulai, L., Calabrese, J.R., Thomas, R., Thompson, M.D., Wilson, B. et al. (2005) Maintenance treatment outcomes in older patients with bipolar I disorder. Am J Geriatr Psychiatry, 13, 305–311.

2. United Nations (2019): Population. https://www.un.org/en/sections/issues-depth/population/index.html. (2019). Accessed 12 Oct 2020

3. Abdul-Hamid, W. K., Lewis-Cole, K., Holloway, F., & Silverman, A. M. (2015). Comparison of how old age psychiatry and general adult psychiatry services meet the needs of elderly people with functional mental illness: Cross-sectional survey. The British Journal of Psychiatry: the Journal of Mental Science, 207(5), 440-443.

4. National Institute for Health and Care Excellence (NICE). Bipolar disorder: assessment and management. 2014. https://www.nice.org.uk/guidance/cg185/chapter/1-Recommendations#managing-bipolar-depression-in-adults-in-secondary-care-2. Accessed 3 Dec 2020.

5. Hein, L., Dols, A., Eyler, L. T (2020) Bipolar disorders in older adults. In: Hantke, N., Etkin, A., O'Hara, R. Handbook of Mental Health and Aging (3rd Edition). Academic Press. Elsevier Inc, p 135-147.

6. Aziz, R., Lorberg, B., Tampi, R.R (2006) Treatments for late-life bipolar disorder. Review Am J Geriatr Pharmacother 4(4):347-64

7. Chen, P., Dols, A., Rej, S., & Sajatovic, M. (2017) Update on the epidemiology, diagnosis, and treatment of mania in older-age bipolar disorder. Curr Psychiat Rep, 19(8), 46.

8. Angst, J., Gamma, A., Sellaro, R., Lavori, P.W., Zhang, H (2003) Recurrence of bipolar disorders and major depression. A life-long perspective. Eur Arch Psychiatry Clin Neurosci, 253(5), 236–40.

For peer review only

For peer review only - http://bmjopen.bmj.com/site/about/guidelines.xhtml

9. Samamé, C., Martino, D.J., Strejilevich, S.A (2013) A quantitative review of neurocognition in euthymic late-life bipolar disorder. Bipolar Disord, 15, 633–44.

10. Rise, I.V., Haro, J., Gjervan., B (2016) Clinical features, comorbidity, and cognitive impairment in elderly bipolar patients. Neuropsych Dis Treat, 12, 1203–13.

11. Beyer J.L., Kuchibhatla, M., Cassidy, F., Krishnan, K.R (2008) Stressful life events in older bipolar patients. Int J Geriatr Psychiatry, 23(12), 1271–5.

12. Nivoli, A.M., Murru, A., Pacchiarotti, Valenti, M., Rosa, A., Hidalgo, Mazzei. D. et al. (2014). Bipolar disorder in the elderly: a cohort study comparing older and younger patients. Acta Psychiatr Scand, 130, 364–373.

13. Kessing, L. V. (2006). Diagnostic subtypes of bipolar disorder in older versus younger adults. Bipolar Disord, 8(1), 56-64.

14. Oostervink, F., Boomsma, M.M., Nolen, W.A. (2009) Bipolar disorder in the elderly: different effects of age and of age of onset. J Affect Disord, 116, 176–183.

15. Jones, S.H., Smith, G., Mulligan, L.D., Lobban, F., Law, H., Dunn, G., et al. (2015) Recovery-focused cognitive-behavioural therapy for recent-onset bipolar disorder: randomised controlled pilot trial. Br J Psychiatry, 206, 58–66.

16. Tyler, E., Lobban, F., Sutton, C., Depp, C., Johnson, S., Laidlaw, K. et al. (2016) Feasibility randomised controlled trial of Recovery-focused Cognitive Behavioural Therapy for Older Adults with bipolar disorder (RfCBT-OA): study protocol. BMJ Open. 6: e010590. doi:10.1136/bmjopen-2015-010590

17. Campbell, M., Fitzpatrick, R., Haines, A., Kinmonth, A.L. (2000). Framework for design and evaluation of complex interventions to improve health. Br Med J, 321, 694–6.

For peer review only

For peer review only - http://bmjopen.bmj.com/site/about/guidelines.xhtml

18. Craig, P., Dieppe, P., Macintyre, S., Michie, S., Nazareth, I., Petticrew, M. (2008). Developing and evaluating complex interventions: the new Medical Research Council guidance. Br Med J, 337, 979–83.

19. Morgan, D. L. (1997). Focus groups as qualitative research (2nd ed.). Thousand Oaks, CA: Sage.

20. Bloor, M., Frankland, J., Thomas, M., & Robson, K. (2001). Focus groups in social research. Thousand Oaks, CA: Sage Publications Inc.

21. Baumgartner, T. A., Strong, C. H., Hensley, L. D. (2002). Conducting and reading research in health and human performance (3rd ed.). New York: McGraw-Hill.

22. Johnson, R. B., Christensen, L. B. (2004). Educational research: Quantitative, qualitative, and mixed approaches. Boston: Allyn and Bacon.

23. Ritchie, J., Spencer, L. (1994) Qualitative data analysis for applied policy research. In: Bryman A, Burgess RG. Analysing Qualitative Data. London: Routledge; p. 173-194.

24. Dixon-Woods, M. (2011). Using framework-based synthesis for conducting reviews of qualitative studies. BMC Med, 9, 39.

25. Furber, C. (2010). Framework analysis: a method for analysing qualitative data. Afr J Midwifery Womens Health 4 (2), 97–100.

26. Gildengers, A.G., Butters, M.A., Chisholm, D., Anderson, S.J., Begley, A et al. (2012). Cognition in older adults with bipolar disorder versus major depressive disorder. Bipolar Disord, 14(2), 198-205.

27. Lala, S. V., Sajatovic, M. (2012). Medical and psychiatric comorbidities among elderly individuals with bipolar disorder: A literature review. J Geriatr Psych Neur, 25(1), 20-25.

For peer review only

28. Tsai, S. Y., Kuo, C. J., Chung, K. H., Huang, Y. L., Lee, H. C. et al. (2009). Cognitive dysfunction and medical morbidity in elderly outpatients with bipolar disorder. Am J Geriat psychiat, , 17(12), 1004-1011.

29. Singh, A., Misra, N. (2009) Loneliness, depression and sociability in old age. Ind Psychiatry J, 18(1), 51-55.

30. Laidlaw, K., Kishita, N., Chellingsworth, M. (2016) A Clinicians guide to: CBT with older people. https://www.uea.ac.uk/documents/246046/18056409/CLINICIANS+GUIDE+TO+CBT+WITH+OLDER+PEOPLE+-+Beck-new.pdf/0adffe3a-2346-4fb2-b0b2-6bad3d20bd47 (2016). Accessed 3 Oct 2020

31. Daley S, Newton D, Slade M, Murray J, Banerjee S (2013). Development of a framework for recovery in older people with mental disorder. Geriatric Psychiatry, 28, (5), 522-529.

32. Gum, A.M., Areán, P.A., Hunkeler, E., Tang, L.M.A., Katon, W., Hitchcock, P. et al. (2006). Depression Treatment Preferences in Older Primary Care Patients. Gerontologist, 46 (1), 14-22.

33. Mead, S., Copeland, M.E. (2000) What Recovery Means to Us: Consumers' Perspectives. Community Ment Health J 36, 315–328.

34. Jones, S.H., Knowles, D., Howarth, E., Lobban, F., Emsley, R. (2021) Mediation analysis of recovery-focused therapy for recent-onset bipolar disorder, J Affect Disord Rep, 5.

35. Knight, R.G., Laidlaw, K. (2009) Translational theory: A wisdom-based model for psychological interventions to enhance well-being in later life. In: Bengtson VL, Gans D, Putney NM, Silverstein M. (Eds.). Handbook of theories of aging (2nd ed). New York: Springer.

For peer review only

Standards for Reporting Qualitative Research (SRQR)

http://www.equator-network.org/reporting-guidelines/srqr/

Title and abstract

	Page
**Title** - Concise description of the nature and topic of the study Identifying the study as qualitative or indicating the approach (e.g., ethnography, grounded theory) or data collection methods (e.g., interview, focus group) is recommended	1
**Abstract** - Summary of key elements of the study using the abstract format of the intended publication; typically includes background, purpose, methods, results, and conclusions	2

Introduction

**Problem formulation** - Description and significance of the problem/phenomenon studied; review of relevant theory and empirical work; problem statement	4
**Purpose or research question** - Purpose of the study and specific objectives or questions	4 & 5

Methods

**Qualitative approach and research paradigm** - Qualitative approach (e.g., ethnography, grounded theory, case study, phenomenology, narrative research) and guiding theory if appropriate; identifying the research paradigm (e.g., postpositivist, constructivist/ interpretivist) is also recommended; rationale**	5
**Researcher characteristics and reflexivity** - Researchers' characteristics that may influence the research, including personal attributes, qualifications/experience, relationship with participants, assumptions, and/or presuppositions; potential or actual interaction between researchers' characteristics and the research questions, approach, methods, results, and/or transferability	8
**Context** - Setting/site and salient contextual factors; rationale**	7
**Sampling strategy** - How and why research participants, documents, or events were selected; criteria for deciding when no further sampling was necessary (e.g., sampling saturation); rationale**	5 & 6
**Ethical issues pertaining to human subjects** - Documentation of approval by an appropriate ethics review board and participant consent, or explanation for lack thereof; other confidentiality and data security issues	5
**Data collection methods** - Types of data collected; details of data collection procedures including (as appropriate) start and stop dates of data collection and analysis, iterative process, triangulation of sources/methods, and modification of procedures in response to evolving study findings; rationale**	6, 7 & 8

For peer review only - http://bmjopen.bmj.com/site/about/guidelines.xhtml

**Data collection instruments and technologies** - Description of instruments (e.g., interview guides, questionnaires) and devices (e.g., audio recorders) used for data collection; if/how the instrument(s) changed over the course of the study	6 & 7
**Units of study** - Number and relevant characteristics of participants, documents, or events included in the study; level of participation (could be reported in results)	8 & 9
**Data processing** - Methods for processing data prior to and during analysis, including transcription, data entry, data management and security, verification of data integrity, data coding, and anonymization/de-identification of excerpts	7 & 8
**Data analysis** - Process by which inferences, themes, etc., were identified and developed, including the researchers involved in data analysis; usually references a specific paradigm or approach; rationale**	7 & 8
**Techniques to enhance trustworthiness** - Techniques to enhance trustworthiness and credibility of data analysis (e.g., member checking, audit trail, triangulation); rationale**	7 & 8

Results/findings

**Synthesis and interpretation** - Main findings (e.g., interpretations, inferences, and themes); might include development of a theory or model, or integration with prior research or theory	10 - 23
**Links to empirical data** - Evidence (e.g., quotes, field notes, text excerpts, photographs) to substantiate analytic findings	10 -23 & supplementary quote table

Discussion

**Integration with prior work, implications, transferability, and contribution(s) to the field -** Short summary of main findings; explanation of how findings and conclusions connect to, support, elaborate on, or challenge conclusions of earlier scholarship; discussion of scope of application/generalizability; identification of unique contribution(s) to scholarship in a discipline or field	21- 25
**Limitations** - Trustworthiness and limitations of findings	3 & 26

Other

**Conflicts of interest** - Potential sources of influence or perceived influence on study conduct and conclusions; how these were managed	27
**Funding** - Sources of funding and other support; role of funders in data collection, interpretation, and reporting	27

For peer review only - http://bmjopen.bmj.com/site/about/guidelines.xhtml

For peer review only

For peer review only - http://bmjopen.bmj.com/site/about/guidelines.xhtml

Focus group topics and content

Focus group 1

1. Icebreaker

Focus group 1 started with an icebreaker (participants went around the room introducing themselves and telling the group an interesting fact about themselves).

2. Recovery approach introduced

The recovery approach was introduced and handed out on a piece of paper, defined as:

"Recovery is being able to live a meaningful and satisfying life, as defined by each person, in the presence or absence of symptoms. It is about having control over and input into your own life. Each individual's recovery, like his or her experience of the mental health problems or illness, is a unique and deeply personal process." (Scottish Recovery Network)

Participants were asked to comment on:

- their thoughts about this quote
- the term 'recovery'
- what recovery meant to them in later life

3. Experience of living with bipolar disorder in later life

Participants were asked to talk about their experience of living with bipolar disorder in later life.

4. Ways of coping with bipolar disorder in later life

Participants were asked about different ways of coping with the condition (including support from relatives and health professionals) in later life.

Focus group 2

1. Introductions

Focus group 2 started with everyone introducing themselves again, with the chance for new participants to tell an interesting fact about themselves.

2. Introducing recovery focused CBT and the stages of therapy

Recovery focused CBT therapy was introduced to the participants and the stages of therapy were discussed:

1. Introducing the recovery approach to clients;
2. Collection of information about current and historical mood and functioning;
3. Meaning and relevance of diagnosis;
4. Identification of recovery-informed therapy goals;
5. Initial formulation of relationships between mood experiences and progress towards recovery goals;

For peer review only

6. Identification and application of CBT techniques to address and facilitate positive coping;
7. Consideration of wider functioning issues in relation to recovery;
8. Development and completion of early warning signs (EWS) plan;
9. Development and completion of recovery plan;
10. Sharing lessons from therapy with key stakeholders.

Participants were asked to comment on:

- whether they thought the approach would be helpful with older adults
- what the strengths and weaknesses of the approach were
- what modifications (if any) would be needed for an older population

2. Session structure

Participants were told that the intervention would be delivered in one-to-one sessions and asked:

- what else might be important to include in therapy sessions for older people
- what might make therapy more accessible and effective for older people

3. Experience of therapy

Participants were asked to comment:

- if they had experience of receiving psychological therapy
- what they had found helpful/ unhelpful when accessing care and support (e.g. CPN, psychiatrist, therapist, relative) in the past
- what help they would want in the future

Focus group 3

1.Introductions

Focus group 3 started with everyone introducing themselves again.

2. Topics

All of the topics above were re-visited in focus group 3 so the three participants who hadn't attended 1 or 2 were able to share their ideas.

For peer review only

Theme	Example quotes
Health and age-related changes in later life	"My concentration isn't very good and the sorts of books I read are different. I can't read anything particularly frightening"<br>"I'm a bit slower but yeah, my memory is OK but like as soon as I get depressed, I get even worse"<br>"When my moods alright so it's slightly elevated, I am … on the ball. I can remember anything…as soon as my mood starts to dip then I start to not…I can't remember what I did the day before"<br>"I think my mood has an effect on my memory…it just gets hard as you get older"<br>"Just my ability to remember things. It's in a limited way now. I put it down to the medication"<br>"I think the only thing I actually do is to listen to the radio and read a certain amount"<br>"We get fed up and we get frustrated and all those things because we can't do what we did"<br>"As you get older there's a lot more…quite often you've got more health issues you've got to deal with...those things can isolate XXX quite a lot. So, when she feels more isolated it can affect mood"<br>"Family members go and social situations change and health deteriorates"<br>"Family structure does change…it's partly what upsets me, I suppose, when I'm in a low. You know, you look back...how many friends and how many family…have gone and you think Christ, you know, I'm the only one here. You don't expect to be the last one."<br>"Such a lot gone. And you just think I miss some of them around me and I must get what I want to do done"

For peer review only

	"The peer thing reduces when you get older... I think the thing that loses, if you get a good peer situation, your identity…when your older, there's a little bit of a problem trying to find your identity".<br>"You're lonely and perhaps not as sharp as you were. So maybe it's a little bit more difficult…maybe we want it but how do we go about it. How do I go about getting involved back in life again"
Experience of BD in later life	"I found that with my manic episodes it's taken me less time to get stable again as the years have gone by. So whether that's an indication that in later life that they will be able to recover quicker and they're not as severe, I don't know."<br>"It counts up to about eight whereas before it went up to nine"<br>"I'm mostly downers. Well if I get in the middle of a picture I'm painting and I'm like enjoying it, I get a high because it's made me really happy…but most of it…I go quiet"<br>"As the depressions have got worse everything pretty much shuts down and I'm depressed"<br>"When I'm on a high I feel like my minds looking up…but when I'm down, I feel like my mind's closed"<br>"I think it loses itself because you can't do the things you did…I do sometimes, and I'm sure everyone does, feels as if well, who am I now? What have I got to contribute?"<br>"Launching yourself out was no problem when you're young, you know, because confidence"<br>"You've got to believe in yourself and you have low self-esteem when you've got bipolar. And people think because you are mentally ill…you need you need to be treated differently"

For peer review only

	"I've got bipolar but I don't go telling other people because I know that, particularly my age group…if you're got to declare a mental illness, it's like well you're not one of them"<br>"It may be that...my psychological problems are sort of focused around get worse as they don't get solved or accepted all the time"<br>"Coming to terms with events that have happened, and you can't understand why that happened and why you did that and why you did this. And with all the embarrassment to cope with"<br>"Yeah, it's regret. Wishing that I'd been, behaved differently or treated somebody differently and that"<br>"If you're doing something that doesn't require a lot of concentration, these things come back to the mind"<br>"I think what's made a difference for my, for my mistakes is that I do have a living faith in God whom I can turn"<br>"What you've got to remember is that if you've got bipolar all your life you've got secrets…you've got personal secrets"<br>"And she kind of knows what she needs to do but there's a greater sense of hopelessness which might be just to do with less life span"
Managing and coping with BD in later life	"There's also been a change in, from my limited experience, that the medications seem to be only a, you know, pop a few pills.... that was the limit of it a while ago but there seems to be other areas being examined ...for example what you are doing"

For peer review only

	"I personally am terrified about what the medication – all the medication I'm on is going to do to me twenty years down the line... what effect it's going to have on my brain"<br>"As you grow older you don't need the amount of medication that you had when you were younger"<br>"Because of the medication being a lot more stable...Ive been able to undertake duties"<br>"I'm able to stabilise myself, sort of consciously change, lifestyle changes, depending on what mood I'm in... act on it quickly and so that I can reach a level ground again"<br>"I think it's a case of learning the illness and being able to manage the illness"<br>"I've painted a lot of pictures which is of course therapeutic"<br>"I think we are capable of doing an awful lot that people think we can't do and therefore we tend to play down our expectations and don't achieve our full potential...if people encourage us, it's surprising wheat we can achieve".<br>"It's about setting out the right goals as well...what older people might be capable of and talking about the sort of things that might be in our own heads individually and whether they are achievable".<br>"If you're having a good weeding session on the allotment, you're concentrating on what you're doing but if you're doing something that doesn't require a lot of concentration, these things come back to mind".<br>"I actually need to be doing at least two things to have a chance of distracting myself. So one way I get through the day when I'm low is to have the radio on but I'm also reading something"

	"I'm in charge of health and safety... and it, well it keeps me mind occupied doesn't it?<br><br>"I would expect health professionals to help you to bond to that community. I would expect them to know what is available to you, to signpost you"<br><br>"As you get older you get more assertive with your professionals"<br><br>"There is a difference now that the professionals treat me with respect and they ask my opinion and how often I want to see them"<br><br>"The younger doctors that I've had dealings with have been brilliant – they've been entirely professional but they've also been informal"<br><br>We're still fighting professionals who don't believe that we're capable of what we truly are and so we don't realise our full potential"<br><br>"A lot of older people they often had a partner that's helped kind of co-helped them co-manage the illness. And when that partner goes, so when my relative died... I didn't realise at the time but he helped modify"<br><br>"I've got a friend now...she phones me up in the morning at eight o'clock and says, how are you and we can be honest with one another"

For peer review only

Experience of recovery in later life	"This recovery we're just talking about... It's something that I look beyond the horizon"<br>"I think the word recovery... you aim for recovery...I think it should be called stability, not recovery"<br>"I don't think you ever recover...you manage it"<br>"Recovery for my therapy team was for me not to go back into hospital... But for me recovery was I really wanted to get another job. It was really important to me"<br>"I'm not sure at my age just to what degree I can move on really as time is flying by… I've been told you can never recover from bipolar"<br>"There is a great difficulty with the word recovery with professionals in the past by they are coming round"<br>"I'm a member of various organisations...I've got an activity every single day to do. And this helps... I'm out and about and I'm mixing with people and this helps enormously".<br>"I think It's possible to be recovered and I reckon that I am recovered now...I have the right medication...and I self-manage. I have a healthy lifestyle and I meet a lot of people"<br>"I've still got a life to live...I can meaningfully contribute to society"<br>"I want to move on from this. There's something better than this. If we haven't got hope, we're just going to wallow"<br>"You wouldn't want to recover if you didn't have hope"

For peer review only

Seeking help in the future	"I was thinking of having time round the therapy... having more time with an old person, sort of fifteen minutes before and fifteen minutes after... just being human together because it's hard doing therapy anyway"<br>"It's about having that personal relationship. It takes two or three sessions to get the confidence"<br>"I think as you get older, you perhaps become quicker at knowing whether you're going to click with somebody"<br>"And we're not as impressable"<br>"We can't be fooled too easily, can we?"<br>"They need to have listening skills. They need to be adapting body language and tone of voice and pitch of voice. They need to be empathic. And they need to be aware of the sort of problems that we face. And they need to get to the cause"<br>"You could raise the gender thing as an explicit issue... find out whether the client has any issues about the gender of the therapist"<br>"The therapist should try to make you feel assertive. Train you to be assertive"<br>"It's part of the therapist's duty to give you higher autonomy and assertiveness so you can fight your corner in a non-aggressive way but one that is assertive"<br>"Therapy focused partly on older age needs to help people to improve competence in whatever area they wanted"<br>"I think that could be a bit of a stumbling block for older people to be able to talk to younger people"

For peer review only

Adapting RfCBT for older people	"I think the idea of fifty minutes is partly that you can have a little bit of chant, you know, at the beginning. Sort of hello, how are you".<br>"When you get older, you know, with sight and problems and hearing problems you just need more resources...things written down... sort of more back up"<br>"She can't take lots of information in really quickly. So more time. Writing things down. Get her to write it down"<br>"Can you break it down...into simple clear language"<br>"Some large sort of text...if it's complicated it could be simplified"<br>"You need to use more images"<br>"It's more interesting to kind of ...engage with and it's easy to kind of, like you said about remembering things you have something to hang on to"<br>"I think a video would be extremely useful...then you can … a disk that you can put in computer and you could watch on the screen an instruction"<br>"If you have it once a fortnight, you've time to put into practice what's been said and what's been taught"<br>"It's quite useful to have a gap...you've learnt some practical strategies...then you've had time to practice on your own...without the weekly backup"<br>"The idea of a short-ish course and then maybe going back in"

For peer review only
